# Not Your Mama’s Ideology (But Close): Modest Generational Differences in Ideological Alignment in Postcommunist Europe

**DOI:** 10.1093/poq/nfag041

**Published:** 2026-06-29

**Authors:** Lenka Hrbková, Tadeas Cely, Matej Jungwirth

**Affiliations:** Assistant Professor, Department of Political Science, Faculty of Social Studies, Masaryk University, Czech Republic; Postdoctoral Researcher, Department of Political Science, Aarhus University, Aarhus, Denmark; PhD Candidate, Department of Political Science, Northwestern University, Evanston, IL, US

## Abstract

This paper examines whether generational change in Central and Eastern Europe (CEE) is associated with shifts in how political attitudes align along the conventional economic–cultural left–right dimension. Because cohorts in the region were socialized under different political–economic systems, CEE provides a setting to assess whether younger generations display patterns of attitude alignment that differ from those of older cohorts. Using pooled European Social Survey data (2004–2023) and original surveys from Czechia, Hungary, and Lithuania, we combine an age–period–cohort analysis of left–right alignment with a network analysis of associations among thirteen salient issues. We find modest generational differences in the early 2000s, but these diminish over time, with all cohorts exhibiting similarly low levels of left–right alignment. Economic and cultural attitudes remain weakly connected, and EU- and foreign-policy issues often form separate clusters. The findings indicate that generational replacement has not produced a shift toward a more conventional left–right organization of belief systems in the region.

Scholars have long debated whether citizens organize their political attitudes in ideologically coherent ways that mirror elite frameworks. Classic studies portrayed the mass public as inconsistent and ideologically unstructured, lacking the constraints and cohesion observed among political elites (Converse 1964; Kinder and Kalmoe 2017). More recent research challenges this view, suggesting that belief systems may exhibit internal structure, but not necessarily along the conventional left–right continuum (Baldassarri and Goldberg 2014; Feldman and Johnston 2014). This shift led to a growing emphasis on bottom-up approaches that empirically examine how individuals organize political attitudes—rather than assuming a predefined ideological structure (Baldassarri and Goldberg 2014; Daenekindt et al. 2017; Barbet 2020; Keskintürk 2022; Van Noord et al. 2025). While the internal structure of belief systems has received increasing scholarly attention, the question of whether such structures evolve across generations—shaped by regime change and substantial institutional reconfiguration, for example—remains underexplored. These debates raise an important question: Do political generations shaped by different institutional contexts organize their beliefs in systematically different ways?

Central and Eastern Europe (CEE) offers a valuable context for examining shifts in belief-system structures, as it consistently exhibits ideological patterns that diverge from those in Western democracies. Malka et al. (2019) and Van Noord et al. (2025) show that CEE citizens frequently exhibit belief-system configurations in which economic and cultural attitudes do not map onto the conventional left–right dimension. Driven by their context, individuals in the CEE region may associate conservative orientations not with defending market hierarchies (as in Western democracies) but with preserving the “ethos of equality” inherited from the previous regime, that is, the expectation that the state should secure a relatively equal economic order (Jami and Kemmelmeier 2021). In this setting, the resistance to change reflects support for maintaining redistributive norms rather than adopting economic liberalism. This produces a configuration in which economic egalitarianism coexists with cultural traditionalism, a well-documented pattern across postcommunist societies (Thorisdottir et al. 2007; Caughey et al. 2019; Malka et al. 2019; Van Noord et al. 2025).

More than thirty years after the collapse of communist regimes, however, the formative environments of citizens in CEE have changed substantially. Individuals who came of age under state socialism were socialized within a distinct institutional and informational system, and these experiences continued to shape attitudes well into the post-transition period (Neundorf 2010; Neundorf and Smets 2016; Pop-Eleches and Tucker 2017). By contrast, younger cohorts have grown up entirely under democratic institutions, market economies, and EU membership. Their socialization differs not only from that of their parents but also from transitional cohorts shaped by the upheavals of the 1990s. These transformations raise the possibility that younger generations might organize political attitudes differently, potentially moving toward belief-system structures that more closely resemble the left–right ideological patterns found in consolidated democracies.

Despite this possibility, we have limited empirical evidence on whether belief systems in CEE have become more ideologically aligned across generations. By alignment, we refer to the extent to which economic and cultural attitudes covary along a shared left–right dimension; disalignment describes weak or negative covariance between these domains without implying internal incoherence. The term signals divergence from a dominant pattern, not a lack of internal structure. If younger cohorts socialized under democratic capitalism developed more integrated ideological structures, we would expect stronger left–right alignment and greater internal constraint among them. Whether such generational restructuring has occurred remains an open question.

We address this question using two complementary approaches. First, we analyze cohort differences in the alignment of economic and cultural attitudes using European Social Survey data from 2004 to 2023. Second, drawing on original survey data from Czechia, Hungary, and Lithuania, we estimate attitudinal networks for each political generation to assess how strongly different issues cluster and whether economic and cultural domains are more tightly linked among younger cohorts. Across both analyses, we find only modest evidence that younger cohorts exhibit stronger left–right alignment or greater internal constraint. Disaligned structures remain widespread, suggesting that generational replacement has not substantially altered the organization of political attitudes in postcommunist democracies.

## The Structure of Political Beliefs

The left–right dimension is analytically useful because it bundles diverse considerations, such as egalitarianism versus hierarchy or openness to change versus tradition, into a simple heuristic widely used by citizens, elites, and spatial models of political competition (Downs 1957; Huber and Inglehart 1995; Mair 2007; Cochrane 2015; Jost 2021; Noël et al. 2021). Yet recent research shows that this model rarely reflects how individuals actually organize their beliefs. Two ideological dimensions usually emerge: an economic axis (redistribution, regulation) and a cultural axis (morality, identity) (Freire 2015; Caughey et al. 2019; Johnston and Ollerenshaw 2020). These dimensions may correlate, but often operate independently (Zumbrunnen and Gangl 2008). They are shaped by distinct psychological traits (Duckitt and Sibley 2010; Fatke 2017) and shape political behavior in different ways (Caprara and Vecchione 2018).

People vary in how consistently their views align across issues. Feldman and Johnston (2014) show that citizens combine economic and cultural attitudes in multiple configurations, not all of which follow the left–right alignment. Similarly, Baldassarri and Goldberg (2014) show that nontraditional configurations, such as economic conservatism paired with cultural liberalism, are common. These findings suggest that ideological constraint does not require left–right alignment, and that belief systems can follow an alternative logic rooted in specific political contexts.

Still, research from Western democracies shows that many citizens hold economic and cultural attitudes that point in a similar ideological direction, although this pattern is not universal (Feldman and Johnston 2014; Klar 2014; Azevedo et al. 2019). The tendency to structure beliefs in ways that align economic and cultural positions with the left–right orientation can be strengthened by individual-level factors, such as political sophistication, psychological needs for certainty, and system-level factors, such as clear elite cues or higher party system polarization (Treier and Hillygus 2009; Gonthier and Guerra 2023). In comparative perspective, this type of positive covariance between ideological domains is found most often in long-standing, institutionalized democracies and highly developed countries (Malka et al. 2019).

In countries with recent authoritarian legacies, fragmented party systems, or weak elite–mass linkages, belief-system structures may differ substantially from the left–right patterns common in Western Europe. Malka et al. (2019) show that in postcommunist countries, economic and cultural attitudes often correlate negatively: Individuals who favor redistribution and state ownership frequently endorse culturally conservative positions on immigration, gender roles, and sexuality. By contrast, in long-established Western democracies, these domains more typically correlate positively, producing the familiar configuration in which economic leftism aligns with cultural liberalism. This “reverse” association in CEE reflects region-specific historical and institutional legacies rather than a deviation from an expected Western norm (Gonthier and Guerra 2023; Van Noord et al. 2025). Consistent with this, Caughey et al. (2019) find that CEE citizens tend to be more economically left-wing and culturally right-wing than their Western counterparts. These findings underscore that ideological organization in postcommunist societies follows a distinct logic, shaped by the legacy of state socialism and the trajectories of post-transition party systems.

## Political Beliefs in Post-Communist Countries

This belief-system divergence has both structural and motivational roots. In Western democracies, conservatism typically involves acceptance of inequality and resistance to change (Jost 2021). In CEE, by contrast, individuals with traditionalist attitudes frequently support economic redistribution (Duriez et al. 2005; Barni et al. 2016; Hadarics 2017). Cross-national work also shows that psychological dispositions associated with left and right in the West map differently onto ideological identities in postcommunist settings (Thorisdottir et al. 2007; Malka et al. 2014). These patterns point to the lasting imprint of authoritarian egalitarianism.

These motivational patterns appear in belief structures. Comparative studies show that political attitudes in CEE are more fragmented and less interconnected than in Western democracies. Individuals link economic and sociocultural views less consistently and combine a wider range of configurations, many of which are weakly represented in party competition. Attitude networks are also thinner and less integrated, suggesting looser ideological organization (Barbet 2020; Keskintürk 2022; Gonthier and Guerra 2023).

The most comprehensive recent study of belief-system structures in Europe, Van Noord et al. (2025), shows that attitudes across Europe rarely form a single left–right dimension. Using associations across 20 issues in 18 countries, the authors identify two broad patterns. One, more common among younger, educated, and Western European citizens, approximates a familiar left–right structure where economic and cultural attitudes move in the same direction. The other, prevalent in CEE, is more fragmented. Beliefs cluster around specific issues, and economic and cultural attitudes often correlate negatively. These cross-pressured structures resemble the alternative configurations described by Baldassarri and Goldberg (2014). Following this logic, we assess whether the weak left–right alignment observed in CEE countries holds across generations or whether younger cohorts socialized in liberal-democratic contexts organize their beliefs differently.

## Socialization Under the New Regime

Postcommunist belief systems often combine cultural conservatism with economic egalitarianism. We argue that this configuration is not fixed but depends on the regime context in which individuals were socialized (Neundorf 2010). Older cohorts were socialized under communism or early transition, which fostered combinations of redistribution support and cultural traditionalism outside standard left–right patterns. Younger cohorts born after 1989 were raised in liberal-democratic, market-oriented environments. This contrast raises the question of whether these different socialization contexts have produced different ways of structuring political attitudes.

The psychological meaning and structure of ideologies vary by context, depending on what social arrangements define the status quo (Jost et al. 2003, 343). While socialism offered its own coherent worldview, the post-1989 order links market inequality with democratic pluralism.

For cohorts socialized under socialism, resistance to change thus meant preserving egalitarian economics alongside cultural traditionalism; for cohorts socialized after 1989, it binds traditional culture to the market order instead. This institutional context shapes a new status quo that may foster belief systems where economic and cultural attitudes align in the same direction—not due to greater sophistication, but because the prevailing regime promotes ideological integration akin to Western patterns.

Early adulthood is widely seen as a critical period for internalizing political norms, and cohorts shaped by shared historical conditions tend to carry distinct orientations throughout life (Inglehart 1977; K. Jennings and Niemi 1978; Neundorf and Smets 2016). Generational differences appear across many domains, including vote choice (Dassonneville 2013), political participation (van der Brug and Kritzinger 2012), and broader attitudes (Down and Wilson 2013). Recent work also finds cohort variation in how citizens link issues to parties, with the youngest Western European voters showing the strongest alignment on immigration, LGBTQ+ rights, environmental protection, and left–right placement (Jocker et al. 2025). These studies underscore that cohort-based variation in political thinking is common. At the same time, research on political socialization highlights the enduring role of parents in shaping children’s worldviews (M. K. Jennings and Niemi 1968; Beck and Jennings 1975; Dalton 1982). These authors argue that generations overlap and blend through parental influence during upbringing. While our data do not allow us to examine parental socialization directly,^1^ we acknowledge that political orientations are shaped by more than generational replacement.

In CEE, cohort differences are particularly likely given that generations were socialized under markedly different political regimes. Existing evidence speaks mainly to voter–party alignment. Walczak et al. (2012) show that younger cohorts exhibit weaker links between issue positions and party preferences, though their analysis relies on 2009 data and does not address how attitudes are structured internally. Our study therefore complements existing work by examining whether economic and cultural attitudes relate differently across cohorts shaped by different regime contexts.

Generational effects on political attitudes in CEE are well documented. Pop-Eleches and Tucker (2017) show that individuals socialized under the old regime remain more skeptical of democratic institutions, more supportive of redistribution, and less favorable to market economies—even decades after transition. Broader orientations also continue to reflect the regime context of early adulthood (Neundorf and Smets 2016). Taken together with the earlier discussion of regime-specific belief structures, this suggests that patterns need not persist. We examine whether those socialized after 1989 organize their political views differently from those raised under socialism and whether younger cohorts display stronger associations between economic and cultural attitudes.

## Political Generations

The significance of the relationship between citizens’ birth year, generational milieu, and political beliefs was canonically articulated nearly 100 years ago by Mannheim (1952). Subsequent research shows that cohorts shaped by shared historical contexts develop distinct and lasting political orientations (Braungart and Braungart 1986; Carlsson and Karlsson 1970; Klecka 1971; M. K. Jennings and Niemi 2014). Following Stoker’s distinction, we treat generations in their political rather than genealogical sense: as cohorts bounded by major social, economic, or political disruptions that reshape everyday political experience (Stoker 2014).

In CEE, two such disruptions define cohort boundaries. The transition from communist rule in the early 1990s created a divide between those socialized under state socialism and those raised in postcommunist democracies (Pop-Eleches and Tucker 2017). A second region-wide transformation occurred with EU accession, which introduced legal and economic harmonization and intensified sociocultural exchange with Western Europe. Crucially, these formative events unfolded at roughly the same time across CEE countries, which enables cross-national comparison without conflating different historical turning points (Grasso 2014).

The study of political generations draws heavily on the theory of impressionable or formative years, developed since Mannheim’s classic work (Mannheim 1952). This perspective holds that experiences in adolescence and early adulthood have disproportionate influence because political identities are still forming (M. K. Jennings and Markus 1984; Plutzer 2002; Bartels and Jackman 2014; Neundorf and Smets 2016). As noted by Delli Carpini (1989), scholars differ in specifying the exact age span of these years. Mannheim (1952) emphasized ages 17–25, which was also the age range used by M. K. Jennings and Niemi (2014); Lambert (1972) then used 18–26 years; and Heberle (1951) resorted to years 20–30. Recent research suggests that durable political learning begins even earlier, during elementary school (Bartels and Jackman 2014). For this reason, in this paper, we define impressionable years as those between 15 and 25 years of age. In doing so, we also follow the recent approach of Grasso (2014).

Foregrounding the fall of the communist regimes and EU accession as the two key historical events in the CEE region in the past half century, we identify three distinct political generations, based on whether or not they were in their impressionable years during these events:

- Pre-Transition: Those born in 1964 or earlier, who did not experience democratic transition during their impressionable years.
- Post-Transition: Those born between 1965 and 1988, who experienced democratic transition or EU accession during their impressionable years.
- Post-EU: Those born after 1988, whose socialization experiences occurred after EU accession.

Primarily, we define the generational boundaries by assessing whether the historical events fall within individuals’ impressionable years (e.g., those born before 1964 were older than 25 in 1989, and thus their early adulthood was over by the time of the democratic transition). For the cross-sectional time-series analysis, we also include two countries with different EU accession years—Bulgaria (2007) and Croatia (2013)—whose domestic politics, however, were influenced by the EU accession process well before they became full members of the Union.^2^

## Methods

We adopt a two-pronged approach, combining large-*N* individual-level analysis with an in-depth study of ideological alignment in a smaller subset of countries but across a larger set of political issues. First, we use European Social Survey (ESS) data from nine Central and Eastern European EU member states^3^ to analyze trends from 2004 to 2023 on four core political issues: *EU integration, LGBT rights, income inequality, and immigration* (see table 1).^4^

**Table 1.** Issue stances from ESS survey and predefined direction.

Label	Issue	Right-wing stance
EU	European unification gone too far	Agree
Immigration	Immigrants make country worse to live	Agree
LGBT	Gays and lesbians free to live	Disagree
Inequality	Government to reduce income differences	Disagree

**Figure 1.**
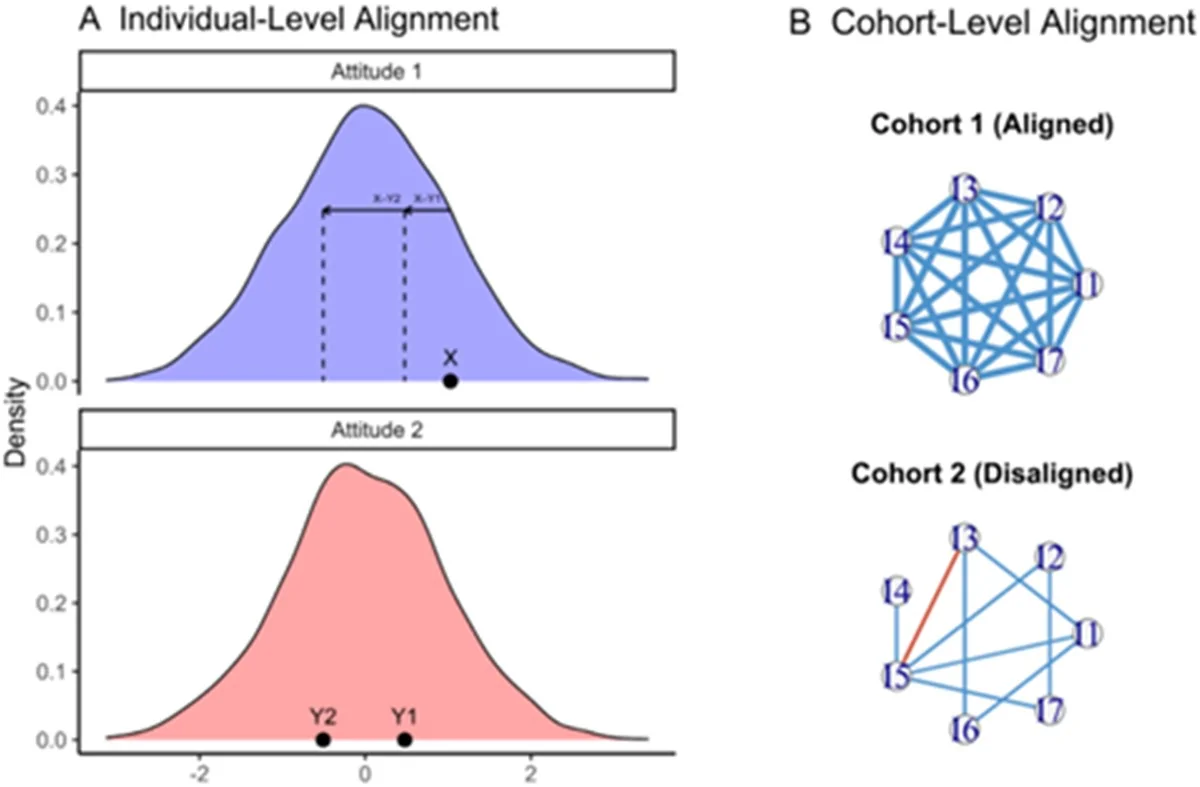
Measurement approaches.

For individual-level analysis, we implement an *age-period-cohort* effects model to examine the alignment of positions across each pair of issues (i.e., six pairs among the four issues identified above: EU integration, LGBT rights, income inequality, and immigration). Alignment is measured through its inverse—the absolute distance between an individual’s positions on the two issues within each pair.^5^  Figure 1A illustrates how this measurement captures alignment. An individual takes a position X on one issue. This position is one standard deviation above the mean and thus liberal relative to the attitudes of others. In two scenarios, they can take a position $Y_{1}$ (0.5 SD above the mean), which indicates alignment (a 0.5 SD distance), as they are liberal on both attitudes. If they take a conservative position $Y_{2}$ (0.5 SD below the mean) instead, they would be disaligned (a 1.5 SD distance between attitudes). The outcome variable is described in Supplementary Material section C.

This approach allows for multivariate analysis that controls for additional socioeconomic differences across generations. We can describe temporal trends in alignment. It also allows for an analysis of heterogeneity in generational effects. The random forest model is summarized in Supplementary Material section E and discussed briefly below.^6^

Second, we compare political generations across a comprehensive set of issues using original survey data collected in late 2024 in Czechia (*N* = 1,381), Hungary (*N* = 1,382), and Lithuania (*N* = 1,330). The online CAWI survey was fielded through Talk Online Panel between November 2 and November 27, 2024, using quota sampling on gender, age, region, size of settlement, and education (best-effort in Lithuania). Full details on recruitment and quality controls appear in Appendix A.

The three countries—representing a stable democracy (Czechia), a case of democratic backsliding (Hungary), and a resilient post-Soviet democracy (Lithuania)—offer a diverse window into the region. The survey includes thirteen salient political issues (see table 2), providing a more granular perspective on ideological alignment in these national contexts.

**Table 2.** Analyzed issue stances and predefined direction.

Label	Issue	Right-wing stance
ec1	State business ownership	Disagree
ec2	Welfare: state responsibility	Disagree
ec3	Graduated taxes	Disagree
gn1	Men better leaders	Agree
gn2	Men job entitlement	Agree
lg1	Adoption by same-sex couples	Disagree
lg2	Same-sex couples - public display	Agree
env1	Environmental spending	Disagree
eun1	EU accession a mistake	Agree
rs1	Support Ukraine at war	Disagree
rs2	Maintain ties with Russia	Agree
im1	Migrants benefit the economy	Disagree
im2	Migrants increase crime	Agree

To test differences in this broader alignment, we follow previous studies (Van Noord et al. 2025; Cely 2025b) in estimating belief networks for individual political generations. For all pairs of political issues within a generation, we compute partial correlations (i.e., the unique association between two issues while holding other attitudes constant). This approach primarily captures aggregate differences between generations rather than variance among individuals (Brandt and Scott Morgan 2022). Figure 1B illustrates how this differs from the previous analysis. Unlike before, alignment is not modeled for each attitude and individual but as the interconnectedness of beliefs within generations.

We then compare networks across generations within each country. Using permutation tests for every correlation (edge) and for the entire network (Epskamp et al. 2018; Van Borkulo et al. 2022), we assess whether a meaningful connection exists between political issues and conduct statistical tests to determine whether alignment differs across generations. For presentation purposes, we do not visualize any connections that do not meet statistical significance.^7^

### Age-Period-Cohort Effects

Age, period, and cohort are in a perfectly deterministic relationship,^8^ making efforts to distinguish between changes in belief alignment due to aging, differing socialization experiences, or contextual influences (e.g., policy moods; Stimson 2018) nearly impossible without further theoretical guidance. Given that this issue cannot be fully resolved by any single statistical solution (Rohrer 2025), we adopt a necessary assumption common to most Age-Period-Cohort (APC) analyses.

The observation period is relatively short for capturing all relevant life stages of political generations (e.g., no individuals socialized after EU accession have yet reached late adulthood), and it lacks a sufficient time span to avoid problems of multicollinearity. Therefore, we assume no statistically significant effect of aging itself on belief alignment, as there is limited theoretical justification to expect beliefs to become more unidimensional merely as a function of age; similarly, recent research finds no consistent aging effect on belief alignment, contrary to popular beliefs (Jocker et al. 2024). Accordingly, we focus on generational differences and period effects. Political generations follow the template presented above, while period is represented by the year of ESS data collection (2004–2023).

**Figure 2.**
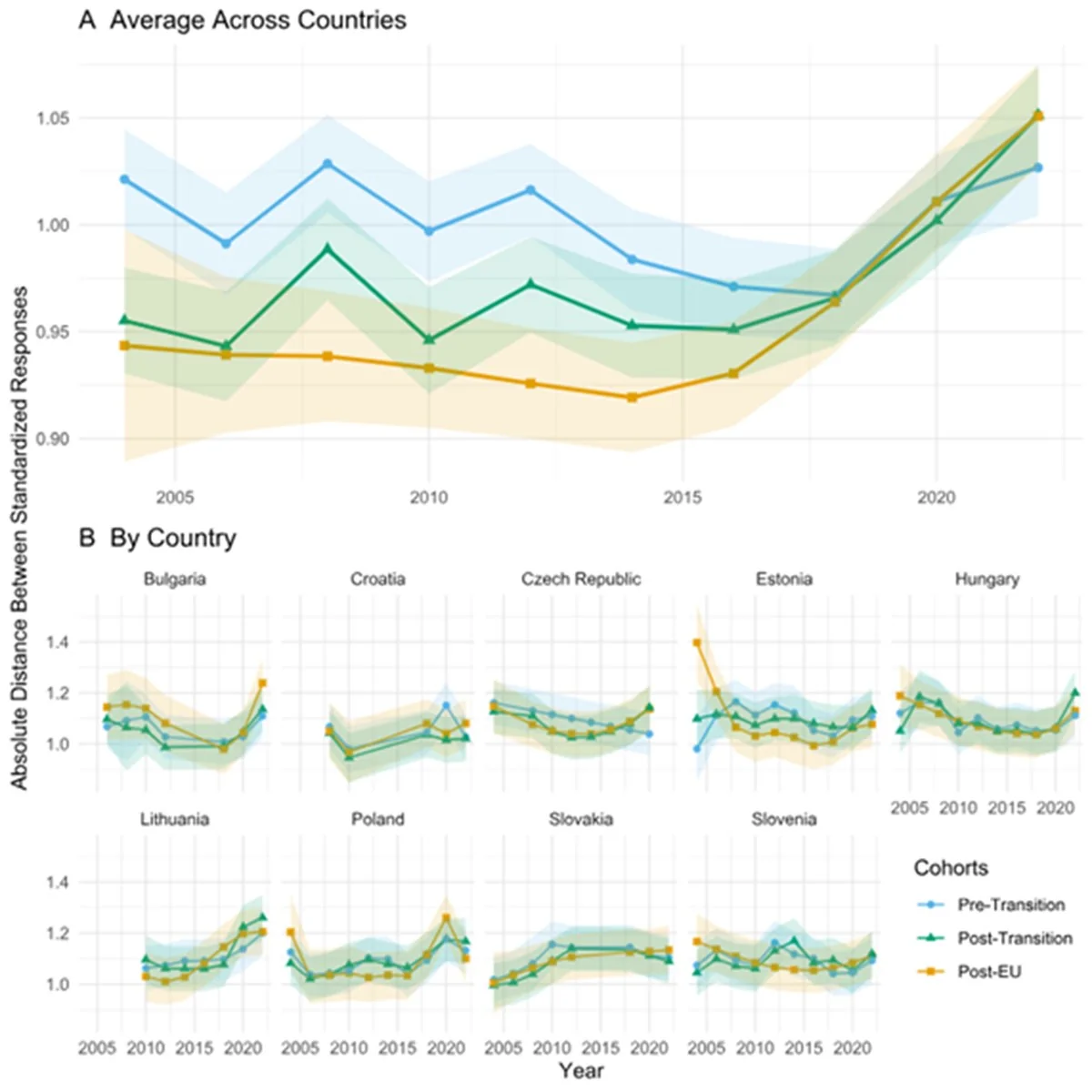
Main cohort differences, with country varying intercepts (A) and by country (B). *N* = 477,438; pairs = 6, countries = 9.

**Figure 3.**
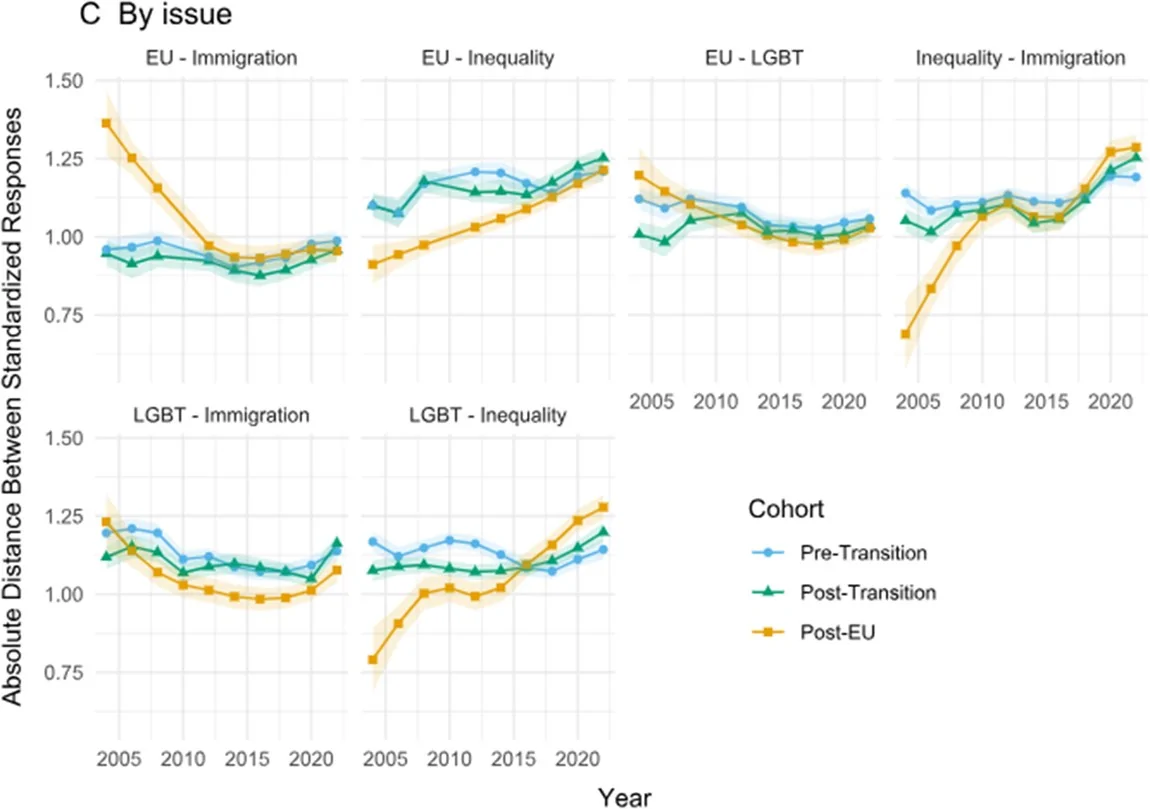
Cohort differences by issue.

To model the alignment of beliefs and following methodological advances in APC analyses (Grasso 2014; Wuttke et al. 2022), we employ a generalized additive mixed model (GAMM) with specific pairs of issues and individual countries included as random intercepts. We estimate the nonlinear effect of survey year and include cohorts as an additional fixed effect. We control for common socioeconomic variables that vary across generations, namely educational attainment and household income, in addition to gender. We model absolute distance—the absolute difference in z-scores between two opinions—through which we capture alignment, understood as its inverse.^9^ For subgroup analyses, we incorporate countries (figure 2B) and individual issue pairs (figure 3) as cross-level interactions in the model.

### Belief Network Analysis

Using networks to measure the interconnectedness of beliefs is a well-established method in public opinion research (see, e.g., Baldassarri and Goldberg 2014; Gonthier and Guerra 2023; Van Noord et al. 2025; Cely 2025b). Networks are estimated using Spearman correlations, as opinions are measured on short ordinal scales.^10^ To assess interconnectedness in a manner consistent with a unidimensional ideological continuum (i.e., alignment with left–right stances as predefined by researchers; see table 2), we employ the measure of *global expected influence* (GEI), which captures the extent to which beliefs are constrained along a predefined ideological direction. Full wording of all items is provided in Supplementary Material section A. GEI differs from alternative metrics such as *global strength*, which does not assume directionality.^11^ Given the 78 edges among the 13 issues, both metrics can theoretically range from zero—indicating complete independence among all issues—to 78, which would imply perfect correlations of one across all issue pairs in the predefined direction. Empirically, we expect substantially lower values, as individual beliefs correlate only weakly in European contexts (Cely 2025a), and some negative associations are also very likely (Malka et al. 2019).

Comparing belief networks allows us to assess two key features: network invariance across generations (whether correlations between individual pairs of issues differ; the *M* statistic) and invariance in *global expected influence*, that is, in overall alignment (the *S* statistic). We take advantage of recent methods that use resampling-based permutation testing of each network and provide tests of statistical significance for each type of invariance.^12^ This approach allows us to determine whether belief networks differ across generations in structure, in alignment, and whether these differences are statistically significant.

Additionally, we provide visualizations of networks to facilitate further inspection. Given the complexity of such networks, we prioritize parsimony, with more fine-grained analyses reserved to Supplementary Material sections F and G. All belief networks are visualized using a fixed layout, ensuring that the same political issues occupy consistent locations across all network plots. This design choice allows for direct visual comparisons between networks and focuses analytical attention on the edges—partial correlations between issue pairs—that constitute the core of our analysis. Edge widths are normalized on a common scale, ranging from 0 (statistically insignificant connections omitted) to the strongest observed partial correlation across the three studied countries (0.54), thereby facilitating meaningful cross-national comparisons of network structure.

Additional details are provided in the Supplementary Material. First, we present centrality metrics for each issue in all belief networks (Supplementary Material section F). Second, we offer an overview of all edge weights (partial correlations) in Supplementary Material section G.

### Results: APC Analysis

In the first step, we consider cohort differences and temporal changes in alignment on four political issues across nine CEE countries using the ESS data. We model changes in absolute distances, where alignment is conceptualized as the inverse of distance. Thus, a reduction in distance can be interpreted as an increase in alignment, measured as the difference in standard deviations between the two standardized opinion scores within each issue pair. Models A and B include varying intercepts by issue pair, and Model A additionally includes a stochastic term for country-level varying intercepts. Model C also includes country-level varying intercepts. Confidence intervals therefore reflect all these additional sources of residual variation.

Main results are presented in figure 2. First, we present average cohort differences in alignment (figure 2A). For most of the studied period, we detect modest yet significantly higher alignment among those socialized under more recent regimes. It is important to emphasize that even several decimal points in sample-wide standard deviations are meaningful given the political heterogeneity of individuals within each generation. For instance, at the start of our analysis in 2004, the marginal mean for distance in the pre-transition generation was 1.02 SDs ($CI_{95}=1.00$–1.04), in the post-transition generation it was 0.96 SDs ($CI_{95}=0.93$–0.98), and in the post-EU generation it was 0.94 SDs ($CI_{95}=0.89$–1.00). This pattern is consistent with modest generational differences. In Supplementary Material section E, we further elaborate on heterogeneity in generational differences using the random forest approach. We show that educational attainment, more than any other covariate, is the primary driver among the individual-level predictors of higher alignment in the youngest cohort.

Generational differences, however, interact strongly with temporal trends (see Supplementary Material section D for tabular representation). We detect nonlinear changes in trends around 2014, which lead to convergence across generations toward higher disalignment across all studied political domains (EU, immigration, redistribution, and LGBT). In the most recent available survey wave, pre-transitional (1.03 SDs, $CI_{95}=1.00$–1.05), post-transitional (1.05 SDs, $CI_{95}=1.03$–1.07), and post-EU generations (1.05 SDs, $CI_{95}=1.03$–1.08) exhibit similar marginal means, indicating a comparable degree of alignment.

To further explore this development, we conduct a country-wide analysis of alignment (figure 2B). Even at first glance, there is considerable heterogeneity across countries, although the general pattern—lower alignment among the pre-transitional generation and convergence among younger generations over time—largely holds. Most notably, the trend toward increasing disalignment beginning around 2016 appears to be nearly universal (at least among younger cohorts), with Slovakia being the primary exception (distance persists at ≈ 1.11 SDs). In a few cases, such as Estonia (1.4 SDs, $CI_{95}=1.25-1.55$) or Poland (1.21 SDs, $CI_{95}=1.06-1.35$), post-EU generations in 2004 are particularly disaligned, contrary to the overall pattern. We elicit potential explanations for this trend in selected cases by examining alignment for individual pairs of issues. Overlapping confidence intervals indicate that generational differences are not significantly pronounced in any single country across all issue pairs, given the smaller sample sizes at the country-by-year level (generally approximately 1,000 respondents, further divided into generations).

Given the different domains from which we draw individual political issues, we also explore generational differences for each of the six individual pairs of issues. This analysis is conducted with country-level varying intercepts. Results are depicted in figure 3. Additional descriptive statistics on inter-item relationships are provided in Supplementary Material section H.

An immediately noticeable pattern is that while all other political generations remain relatively stable (though note the increasing disalignment between economic and immigration beliefs beginning around 2016 across all generations), the alignment among post-EU generations is considerably more turbulent. Growing distances on some issue pairs counteract increasing alignment on others, while the overarching trend is one of convergence with the other generations. Another notable feature is the nonlinearity of these changes, with the steepest shifts occurring before 2010, when the aftermath of the economic crisis fully materialized, and again around 2016, following the so-called EU refugee crisis of 2015.

While the post-EU generation generally converges with older cohorts through disalignment, the youngest cohort is more disaligned on pairs involving EU unification and immigration—and, to a lesser extent, LGBT attitudes. This likely reflects how the EU-unification item was interpreted around Eastern enlargement: Respondents with limited experience of the pre-accession harmonization process may have understood it mainly as referring to enlargement itself. For those socialized during this period, these formative perceptions created a temporary disconnect between EU-related and other attitudes in the early 2000s.

The final observation that stands out is the extent to which all other attitudes were initially aligned with economic beliefs (here measured as support for reducing income differences) among the post-EU generation. This provides some support for the argument of Malka et al. (2019), who link CEE exceptionalism to the postcommunist legacy, although this pattern does not extend to those who grew up during the 1990s. Moreover, the divergence of younger cohorts diminishes over time. Nevertheless, in 2004, the marginal mean for the post-EU generation was 0.91 SDs ($CI_{95}=0.85$–0.97) for *EU–Inequality* pair (contrast to pre-transition: 1.10, $CI_{95}=1.07$–1.13; post-transition: 1.10, $CI_{95}=1.06$–1.14), 0.69 SDs ($CI_{95}=0.58$–0.80) for *Inequality–Immigration* (pre-transition: 1.14, $CI_{95}=1.11$–1.17; post-transition: 1.05, $CI_{95}=1.01$–1.09), and 0.79 SDs ($CI_{95}=0.69$–0.89) for *LGBT–Inequality* (pre-transition: 1.17, $CI_{95}=1.14$–1.20; post-transition: 1.08, $CI_{95}=1.04$–1.11). This substantial cohort difference gradually disappeared over time, rendering the economic attitudes of the post-EU generation as disaligned with social attitudes as those of preceding generations.

## Results: Belief Networks in Three Contexts

While the APC analysis reveals only modest generational differences in belief alignment across the CEE region, the ESS surveys cover only a limited range of issues. To assess the internal structure of belief systems—and whether younger cohorts differ in alignment—we estimate and compare belief networks for political generations in three CEE countries: Czechia, Hungary, and Lithuania. This provides a more comprehensive view of cross-domain associations. Because sample size affects how many edges are identified as meaningful, fewer edges among the youngest generation do not necessarily signal lower alignment; GEI captures these connections. We therefore focus on major differences relevant to interpreting our findings.

Before turning to specific cases, we provide an overview of generational differences in belief networks and their alignment across the three studied contexts. These results are summarized in table 3. Our findings reaffirm that, in the most recent survey year (2024), generational differences remain limited.

**Table 3.** Overview of test statistics.

		Network		Alignment			
Country	Comparison	*M*	*p*-value	GEI 1	GEI 2	*S*	*p*-value
Hungary	Post-EU x post-transition	0.17	0.12	3.65	3.99	0.34	0.37
Hungary	Post-EU x pre-transition	0.26^a^	< 0.001^a^	3.65	3.47	0.18	0.59
Hungary	Post-transition x pre-transition	0.19^a^	0.03^a^	3.99	3.47	0.52	0.14
Czechia	Post-EU x post-transition	0.18	0.07	3.36	3.52	0.16	0.61
Czechia	Post-EU x pre-transition	0.24^a^	< 0.001^a^	3.36	3.40	0.04	0.91
Czechia	Post-transition x pre-transition	0.11	0.75	3.52	3.40	0.12	0.67
Lithuania	Post-EU x post-transition	0.17	0.09	3.24	3.57	0.33	0.36
Lithuania	Post-EU x pre-transition	0.22^a^	0.02^a^	3.24	2.94	0.30	0.50
Lithuania	Post-transition x pre-transition	0.18	0.08	3.57	2.94	0.63	0.12

a symbol indicates statistical significance.

Focusing on the main outcome—generational differences in alignment—we find very similar levels of alignment across generations within each country. Across contexts, the post-transition generation exhibits the highest alignment, with the post-EU generation lagging only slightly behind. However, these differences never reach statistical significance, which prevents us from reaching any conclusions with certainty. Substantively, the differences are also small, with *S* values ranging between 0.04 and 0.63, equivalent to a shift of roughly three correlations (out of 78) moving from weak to moderately strong (see Supplementary Material section G for an overview of typical correlation magnitudes).

A second question is whether belief networks differ across generations in ways other than overall alignment. Although networks are largely invariant across most comparisons, several notable exceptions emerge. In all three countries, post-EU generations differ significantly from those socialized before democratic transitions, and in Hungary the pre-transition generation also differs—though less strongly—from those who grew up in the 1990s. The visualizations below highlight where these differences arise and illustrate cross-national variation in belief-network structures.

### Czechia

**Figure 4.**
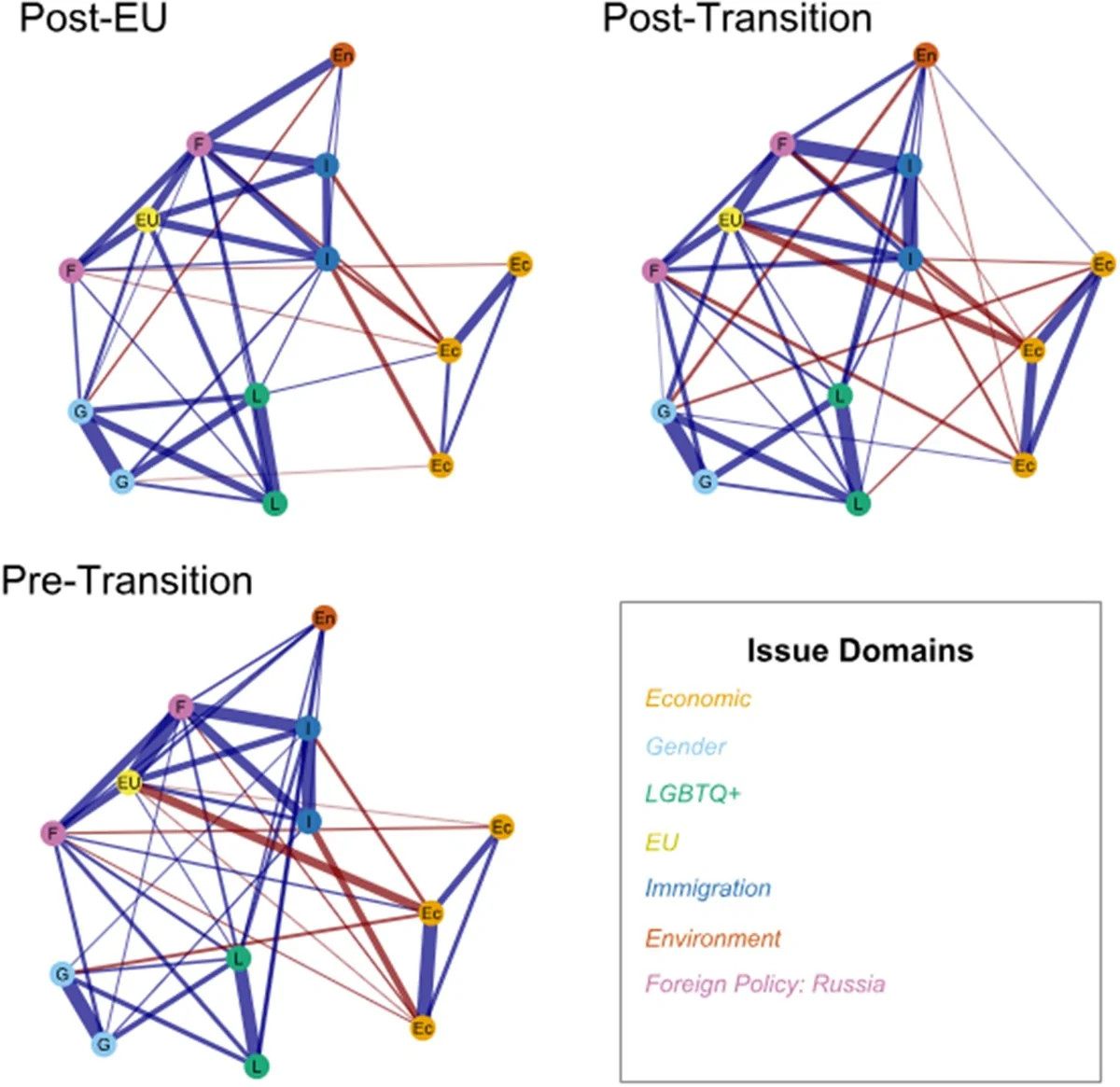
Generational differences in networks of beliefs: Czechia.

In the first example, it is primarily the oldest and youngest generations that differ. Besides an even deeper disconnect between economic and social beliefs—already rather weak among older generations—the belief networks of the post-EU generation in Czechia are shaped primarily by their evaluation of EU accession, which is less aligned with other beliefs. Several of these differences are evident in figure 4. The negative association between economic and social issues is more pronounced in the pre-transitional generation, whereas among the youngest generation these beliefs are largely unassociated. A prominent case is the negative attitude toward EU accession, which correlates with left-wing attitudes on the welfare state in the post-transition (*–0.18*) and pre-transition (*–0.20*) generations, but is much weaker and statistically nonsignificant in the post-EU generation.

A second difference concerns attitudes toward aid to Ukraine, which are somewhat less tied (*0.18*) to the EU accession item than in older generations (post-transition: *0.26*; pre-transition: *0.35*), and somewhat more connected to environmental attitudes (post-EU: *0.21*; post-transition: *0.11*; pre-transition: *0.05*). Overall, however, belief systems are largely invariant across generations.

### Hungary

**Figure 5.**
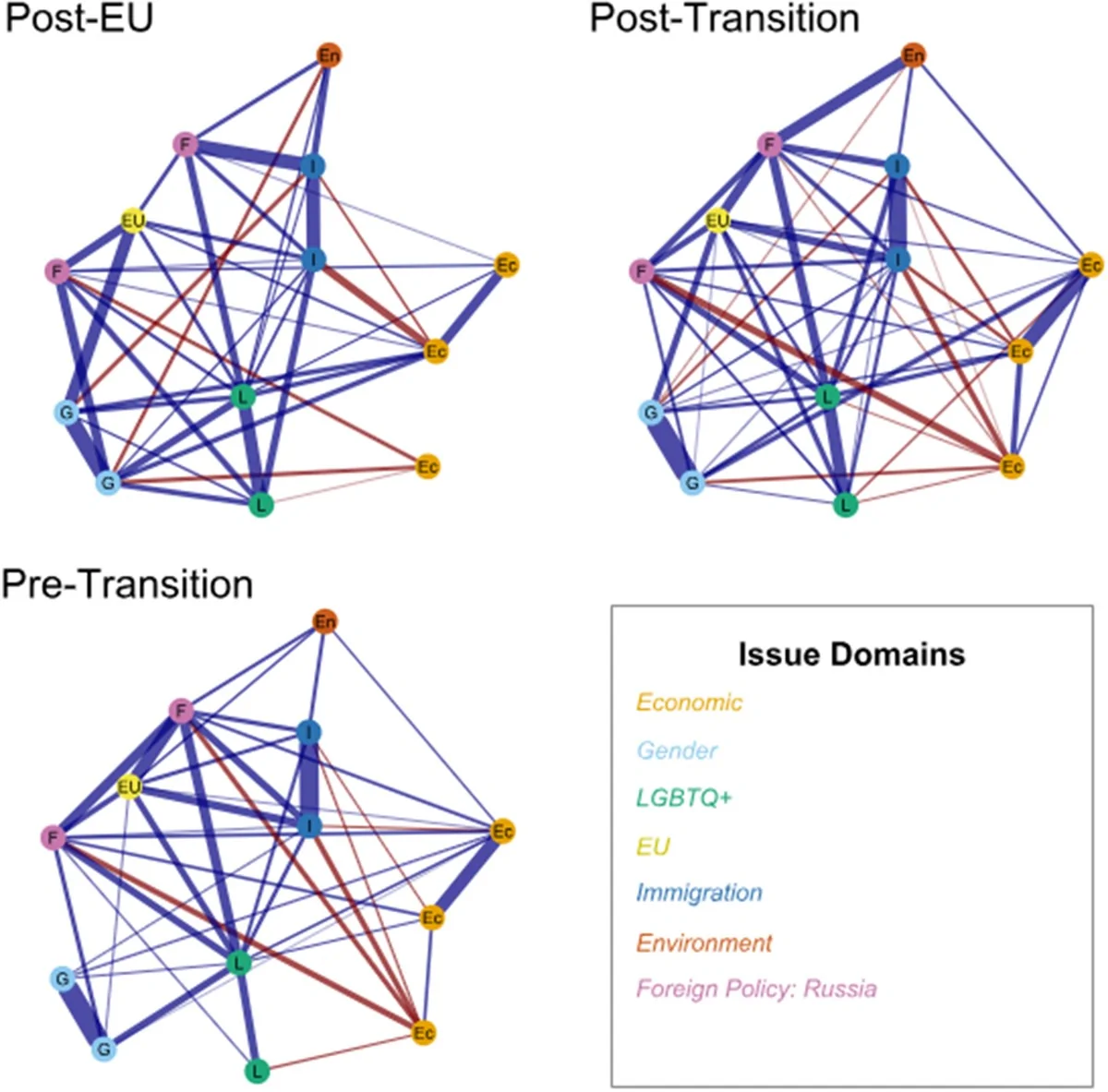
Generational differences in networks of beliefs: Hungary.

In the Hungarian case, both younger generations differ significantly from the oldest one, even if not in alignment. Two focal issues drive this divergence: gender issues and the Russia-Ukraine conundrum (figure 5). First, gender-progressive attitudes are more strongly aligned with rejecting dialogue with Russia in the post-EU generation than in the others (*0.20*; post-transition: *0.06*; pre-transition: *0.08*), and both younger cohorts also link these attitudes more closely to positive evaluations of EU accession (post-EU: *0.26*; post-transition: *0.14*; pre-transition: *nonsignificant*). Aid to Ukraine is likewise more connected to the view that immigrants benefit the economy in the younger cohorts (post-EU: *0.31*; post-transition: *0.15*; pre-transition: *0.09*), a pattern that may reflect attitudes toward Ukrainian refugees.

Finally, while in both the post-transition and pre-transition generations the question of government ownership of business is linked to other economic views and correlates negatively with social views, this association is far weaker—and largely disconnected—among the post-EU cohort. In the Hungarian case, it is also noteworthy that economic issues generally do not correlate negatively with social issues, instead correlating positively or remaining largely dissociated from them.

### Lithuania

**Figure 6.**
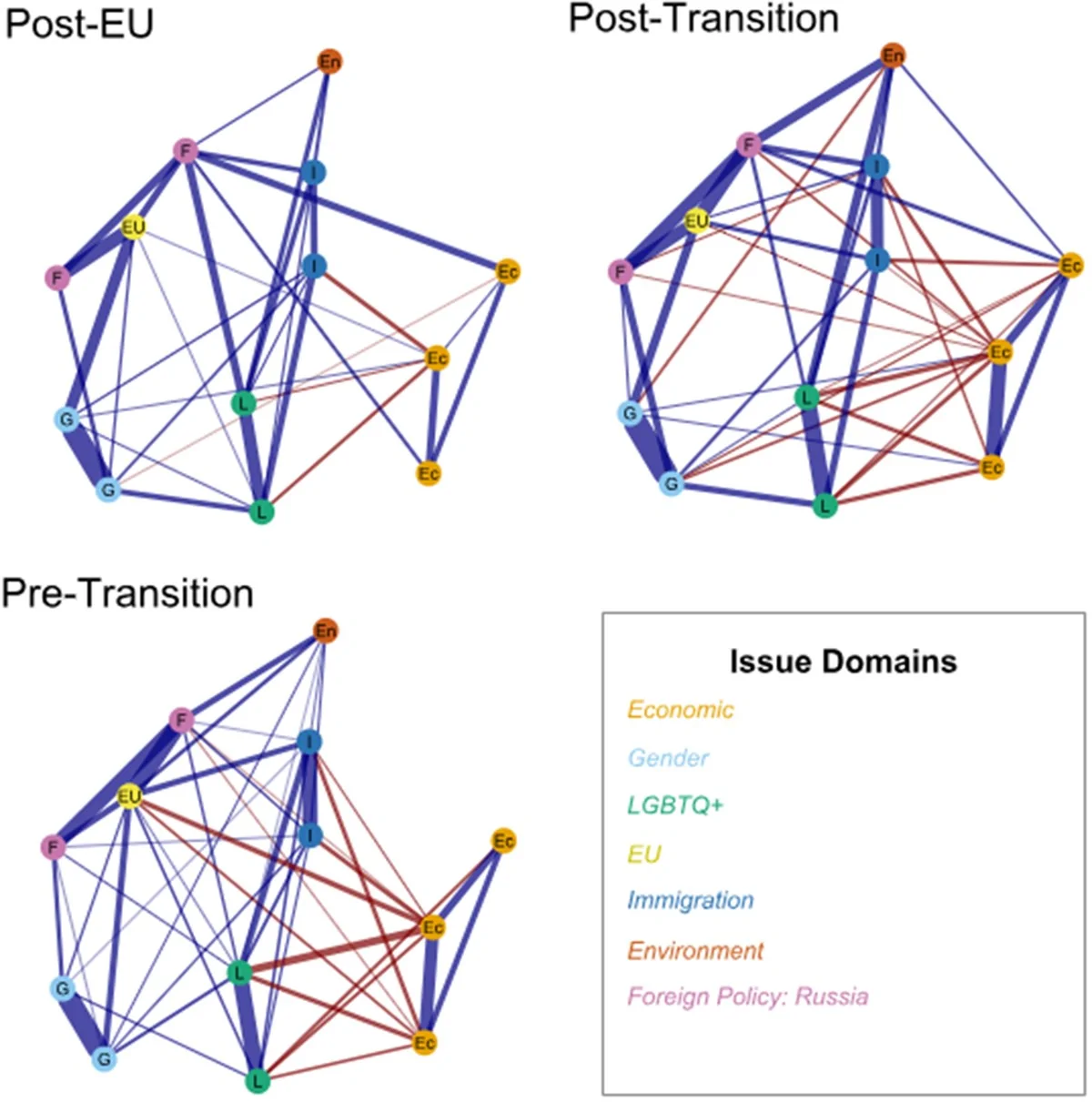
Generational differences in networks of beliefs: Lithuania.

Finally, in the Lithuanian case, we detect significant differences in the configuration of beliefs between the youngest and oldest generations. The primary distinctions concern the alignment of international issues with domestic ones (figure 6). Younger, post-EU Lithuanians connect their views on Ukraine somewhat more strongly to taxation (*0.15*; post-transitional: *0.09*; pre-transitional: *nonsignificant*) and to adoption by same-sex couples (*0.16*; post-transitional: *0.07*; pre-transitional: *0.04*), thereby linking domestic and international issues.

Evaluations of EU accession are also more weakly aligned with support for aid to Ukraine in the post-EU generation (*0.14*) than in older cohorts (post-transitional: *0.31*; pre-transitional: *0.36*), yet they are more strongly associated with views on maintaining diplomacy with Russia (post-EU: *0.30*; post-transitional: *0.27*; pre-transitional: *0.12*). Finally, unlike in older generations—where economic and social domains are weakly negatively correlated—economic issues are largely unassociated with social issues in the post-EU generation.

## Conclusions

In this study, we examined whether younger cohorts in Central and Eastern Europe, particularly those raised in postcommunist democracies, have developed belief systems more aligned along the left–right continuum than older generations. Across most domains—especially those linking economic to cultural or migration attitudes—the youngest cohorts were initially somewhat more aligned, but this advantage was small, limited to the 2000s, and absent for EU-related issues. Over time, these differences dissipate, with all generations converging toward similar patterns of disalignment. These trajectories do not support expectations that economic and cultural right-wing attitudes increasingly converge under democratic and market-oriented socialization. Instead, period-wide influences dominate, and national trajectories diverge, indicating that belief alignment in CEE does not follow a single generational path.

Network analyses of thirteen issues in Czechia, Hungary, and Lithuania corroborate this pattern. Overall alignment is similar across cohorts, and generational differences arise mainly in network structure rather than in the degree to which attitudes map onto a left–right axis. Economic and cultural domains do not crystallize into a shared ideological dimension. Although Hungary shows tighter clustering on a few issues—gender, Russia, and EU—these patterns remain context-specific. Younger cohorts do not exhibit stronger ideological integration; in Czechia and Lithuania, cross-domain ties are even weaker than among older cohorts.

Our findings align with research showing that left–right self-placement is only weakly informative in CEE (Dassonneville and McAllister 2025). Even when examining attitudes directly, economic and cultural domains do not align into a coherent ideological structure, and younger cohorts are no more integrated than their parents. The limited usefulness of left–right labels therefore reflects the structure of mass beliefs rather than a lack of sophistication. Our results indicate that major institutional changes—democratic consolidation, market reforms, and EU accession—have not reorganized public belief systems in CEE; earlier patterns persist, and younger cohorts show only modest movement toward alignment, with economic and cultural attitudes continuing to diverge from a conventional left–right structure (Malka et al. 2019; Van Noord et al. 2025).

Belief systems in CEE countries are not fully unstructured: Citizens cluster attitudes that “go together,” with economic issues grouping consistently and cultural ones more weakly. What is largely absent is covariation across these domains that would produce a left–right ideological structure. We cannot determine from our data whether this reflects limited awareness of which issues typically align, or a conscious preference for such patterns, but the observed disalignment does not appear to be a rejection of a known ideological mapping (Groenendyk et al. 2023). Instead, it reflects the fact that the meaningful associations available in the CEE context differ from those implied by ideological schemas prevalent in Western democracies.

National political trajectories further shape belief-system organization. In Lithuania, Russia-related attitudes remain central (Valentinavičius 2025). In Hungary, links between gender, Russia, and EU issues reflect long-term politicized conflict (Guasti and Bustikova 2023). Czechia displays a more diffuse pattern in which EU and foreign-policy attitudes are only weakly tied to economic or cultural domains. These country differences limit the extent to which generational change produces convergent ideological structures across the region.

Our analysis has several limitations. Both parts of the study are correlational and cannot identify causal effects, describing associations rather than underlying mechanisms. The APC models estimate cohort differences but, because they omit an explicit age term, cannot fully separate generational from life-cycle effects; alignment estimates also depend on the available attitudinal items. The network analysis is descriptive, capturing how issues connect but not why these links arise. Finally, we examine only three countries, and broader coverage would help assess generalizability.

Taken together, our findings suggest that belief systems in CEE remain in flux. Changes in their organization are gradual and uneven, shaped more by contextual dynamics than by generational replacement alone. Rather than converging toward a unified ideological structure, patterns of alignment remain fragmented and context dependent.

## Supplementary Material

nfag041_Supplementary_Data

## Data Availability

Replication data and documentation are available at https://doi.org/10.7910/DVN/PPWBKF.

## Footnotes

1 To comprehensively evaluate the interaction of parental influence and generational differences, we would need high-quality longitudinal panel data for two or more generations and track political beliefs and attitudes of both parents and their children over time.

2 Bulgaria’s accession to the EU was secured through the 2005 Treaty of Accession, and Croatia had already applied to join the EU in 2003. Consequently, we apply the same temporal boundaries in these two cases as well.

3 These include: Bulgaria, Croatia, Czechia, Estonia, Hungary, Lithuania, Poland, Slovakia, and Slovenia. Other CEE countries, such as Serbia and Romania, were not included due to their insufficient coverage across the ESS rounds. See Supplementary Material section B for detailed overview of ESS round coverage of the nine CEE countries included in the analysis.

4 Analyses use listwise deletion for cases with item-missing data.

5 It is calculated as: ${\text{Distance}}_{ij}=|x_{i}-x_{j}|$, where $x_{i}$ and $x_{j}$ denote the positions on issues i and j, respectively. Given that opinions were recorded using scales of varying lengths, all responses were standardized; consequently, we use z-scores rather than raw values.

6 This comes at the expense of excluding several political issues, some of which may be crucial for certain generations and could pose a threat to the robustness of our argument. This limitation is addressed in the second analysis.

7 Significance is determined using the EBICglasso method (Friedman et al. 2008; Foygel and Drton 2010). The LASSO (least absolute shrinkage and selection operator) method identifies substantial connections in the network. The EBIC (extended Bayesian information criterion) serves as a tuning parameter to determine the degree of regularization required given the model selection criterion (e.g., sample size).

8 This is also known as the “identification problem”—once we know two of the three, the third can be unequivocally determined. For example, Age can be computed as Age=Period-Cohort, where Period is the year of observation and Cohort is the year of birth.

9 Opinion scales were recoded so that higher values reflect a left-wing or liberal stance.

10 All opinions in our data collection are measured on five-point scales.

11 Global expected influence is calculated as$\text{Global}\;\text{Expected}\;\text{Influence}=\sum_{i}\left(\sum_{j}w_{ij}\right)$, where $w_{ij}$ is the weight between nodes i and j. Global strength is $\text{Global}\;\text{Strength}=\sum_{i}\left(\sum_{j}\left|w_{ij}\right|\right)$, summing the absolute edge weights; thus, negative correlations are not subtracted from positive ones.

12 Pairwise comparisons between generations were performed using the *NetworkComparisonTest* package (Van Borkulo et al. 2022).

## Appendix A. AAPOR-Required Disclosure Elements

### Data Sources

The study is based on original cross-sectional survey data collected in three Central and Eastern European (CEE) countries and on European Social Survey data, waves 2004–2023 (see Home | European Social Survey). Country and wave coverage is presented in the Supplementary Material. We further discuss the original CEE surveys.

### Data Collection Strategy

Data were collected through a survey administered in Czech, Lithuanian, and Hungarian. The survey was fielded in a single wave in November 2024 using a Qualtrics questionnaire.

### Research Sponsor and Conductor

This research was funded by the Czech Science Foundation (GA22-33158S). The study was designed and programmed by the research team from Aarhus University, Masaryk University, and Northwestern University. Data collection was carried out by Talk Online Panel, which administered the questionnaire through its online respondent panels in Czechia, Hungary, and Lithuania.

### Measurement Tools/Instruments

Survey questions and response options from both the original survey and ESS are presented in Supplementary Material section A.

### Population Under Study

The target population was the adult (18 and older) general public in Czechia, Hungary, and Lithuania.

### Methods Used to Generate and Recruit the Sample

Recruitment relied on the nonprobability online respondent pools maintained by Talk Online Panel s.r.o. in all three surveyed countries. Talk Online Panel operates opt-in online panels comprising approximately 1.3 million users across 24 countries. Samples drawn from these panels are nonprobability samples based on the existing pool of registered panelists.

Panel members are recruited through diversified online channels, including media partnerships, marketing collaborations, social media outreach, digital advertising, newsletters, search-engine marketing, and registrations collected during other surveys conducted by affiliated research agencies. During the registration process, multiple quality-control procedures are used to validate user information. Ongoing data-quality safeguards include random email or telephone verification checks, limits on the number of surveys panelists can complete, and periodic requests to update profile information. Panelists receive no more than six invitations per month, and a cooldown system prevents repeated invitations within short intervals.

In each country, sample selection followed quotas for gender, age, education, region, and settlement size. In Lithuania, quotas for education were achieved on a best-effort basis.

Participants were compensated through Talk Online Panel’s points-based reward system. Panelists accrue points for participating and can redeem them for locally available rewards, including cash payments, shopping vouchers, magazine subscriptions, or charitable donations.

### Method and Mode of Data Collection

Data were collected through computer-assisted web interviewing (CAWI). The questionnaire was programmed in Qualtrics and distributed to respondents via the Talk Online Panel platform.

### Dates of Data Collection

Data collection took place between November 2, 2024, and November 27, 2024.

### Sample Sizes

In each country, 1,400 complete responses were targeted.

### Data Weighting

All analyses used unweighted data.

### How the Data Were Processed and Procedures to Ensure Data Quality

The survey employed an attention check. Only respondents who passed this check were permitted to complete the survey. Duplicate responses linked to a single panelist profile or IP address were removed.

### Panel Description

Panel membership is managed through a structured registration and verification process, including a triple opt-in system, automated duplicate checks, antibot measures, and SMS-based two-factor authentication to ensure that participants are real and unique. Panelists receive regular invitations but are limited to a maximum of six survey invitations per month, with a cooldown period between invitations to prevent over-participation. Participation history, profile completeness, and response quality are monitored continuously, and low-quality respondents are warned or removed through a formal “bad quality” review system. Panel profiles are updated at least annually, and members can revise their data at any time. These procedures support ongoing panel maintenance, ensure respondent quality, and manage attrition within the access panel framework.

### Dispositions or Response or Participation Rates

Response and participation rates are proprietary information not disclosed by the panel vendor.

### Measurement and Model Specification

All measurements and model specifications are disclosed in the methods section of the manuscript and in the Supplementary Material.

### General Statement Acknowledging Limitations of the Design and Data Collection

A detailed discussion of the limitations of the study design and data is provided in the conclusions section of the manuscript.
