# Supplementary material for: Not Your Mama’s Ideology (But Close): Modest Generational Differences in Ideological Alignment in Postcommunist Europe
Source: Public Opin Q. 2026 Jun 29;90(SI):1084–113. doi: 10.1093/poq/nfag041 (PMC13553006; doi:10.1093/poq/nfag041)
Supplement: Supplementary file 1 [file nfag041_supplementary_data.pdf]

Not Your Mama's Ideology (But Close): Modest Generational Differences in Ideological Alignment in Post-Communist Europe

Lenka Hrbkova, Masaryk University[1]

Tadeas Cely, Aarhus University

Matej Jungwirth, Northwestern University

Supplementary Material

Contents

A. Survey Wording 3
B. ESS Wave Coverage in CEE Countries 4
C. Alignment: Outcome Variable Description 5
D. Models in Tabular Form 6
Figure 2A 6
Figure 2B 6
Figure 3 7
E. Heterogeneity in Cohort Differences: Random Forest 9
F. Centrality Measures for Belief Networks 12
Why Strength as a Measure of Centrality? 12
G. Belief Networks - Edge Weights 14
Czechia: Post-EU 14
Czechia: Post-Transition 14
Czechia: Pre-Transition 14
Hungary: Post-EU 15
Hungary: Post-Transition 15
Hungary: Pre-Transition 15
Lithuania: Post-EU 16
Lithuania: Post-Transition 16
Lithuania: Pre-Transition 16

[1] Correspondence should be addressed to Lenka Hrbkova, hrbkova@mail.muni.cz.

H. ESS: Inter-Item Alignment Descriptives 17

A. Survey Wording

The following items were part of an original survey fielded in the Czech Republic, Hungary and Lithuania in late 2024. Respondents were asked to express their attitudes toward selected issues using predefined 5-point scales (1 = Strongly disagree, 5 = Strongly agree).

Issue Attitudes The following section asked respondents about their opinions on selected political issues. Respondents indicated the extent to which they agreed or disagreed with each statement on a 5-point Likert scale: 1 = Strongly disagree 2 = Disagree 3 = Neither agree nor disagree 4 = Agree 5 = Strongly agree

Economic Attitudes **issues_econ1** The state should retain ownership of a substantial share of businesses and enterprises that are currently state-owned. **issues_econ2** The state should take greater responsibility to ensure that everyone is cared for. **issues_econ3** People with higher incomes should pay a higher percentage of their income in taxes than those with lower incomes.

Gender Attitudes **issues_gender1** All things considered, men make better political leaders than women. **issues_gender2** When jobs are scarce, men should have more right to a job than women.

LGBT Attitudes **issues_lgbt1** Gay men and lesbians should have the same right to adopt children as heterosexual couples. **issues_lgbt2** Gay men and lesbians should not display their sexual orientation in public.

European Union **issues_EU1** The Czech Republic's accession to the European Union was a major and fundamental mistake.

Immigration **issues_immigr1** In general, immigrants are beneficial to the Czech economy. **issues_immigr2** Immigrants cause an increase in crime in the Czech Republic.

Environmental Attitudes **issues_environment** I would be willing to give up part of my income if I were sure that the money would be used to prevent environmental pollution.

Russia **issues_russia1** Our country should support Ukraine in regaining lost territory. **issues_russia2** Our country should continue to maintain diplomatic relations with Russia, even under the current circumstances.

The ESS survey items used to measure belief alignment in APC analysis:

euftf European Union: European unification go further or gone too far 0 (Unification already gone too far) – 1 – 2 – 3 – 4 – 5 – 6 – 7 – 8 – 9 – 10 (Unification go further)

freehms Gays and lesbians free to live life as they wish 1 = Agree strongly 2 = Agree 3 = Neither agree nor disagree 4 = Disagree 5 = Disagree strongly

gincdif Government should reduce differences in income levels 1 = Agree strongly 2 = Agree 3 = Neither agree nor disagree 4 = Disagree 5 = Disagree strongly

imwbcnt Immigrants make country worse or better place to live 0 (Worse place to live) – 1 – 2 – 3 – 4 – 5 – 6 – 7 – 8 – 9 – 10 (Better place to live)

B. ESS Wave Coverage in CEE Countries

Table B1: *Country Participation by ESS Round*

Country	Round 2 (2004)	Round 3 (2006)	Round 4 (2008)	Round 5 (2010)	Round 6 (2012)	Round 7 (2014)	Round 8 (2016)	Round 9 (2018)	Round 10 (2020)	Round 11 (2023)
BG	×	✓	✓	✓	✓	×	×	✓	✓	✓
CZ	✓	×	✓	✓	✓	✓	✓	✓	✓	×
EE	✓	✓	✓	✓	✓	✓	✓	✓	✓	✓
HR	×	×	✓	✓	×	×	×	✓	✓	✓
HU	✓	✓	✓	✓	✓	✓	✓	✓	✓	✓
LT	×	×	×	✓	✓	✓	✓	✓	✓	✓
PL	✓	✓	✓	✓	✓	✓	✓	✓	✓	✓
SI	✓	✓	✓	✓	✓	✓	✓	✓	✓	✓
SK	✓	✓	✓	✓	✓	×	×	✓	✓	✓

C. Alignment: Outcome Variable Description

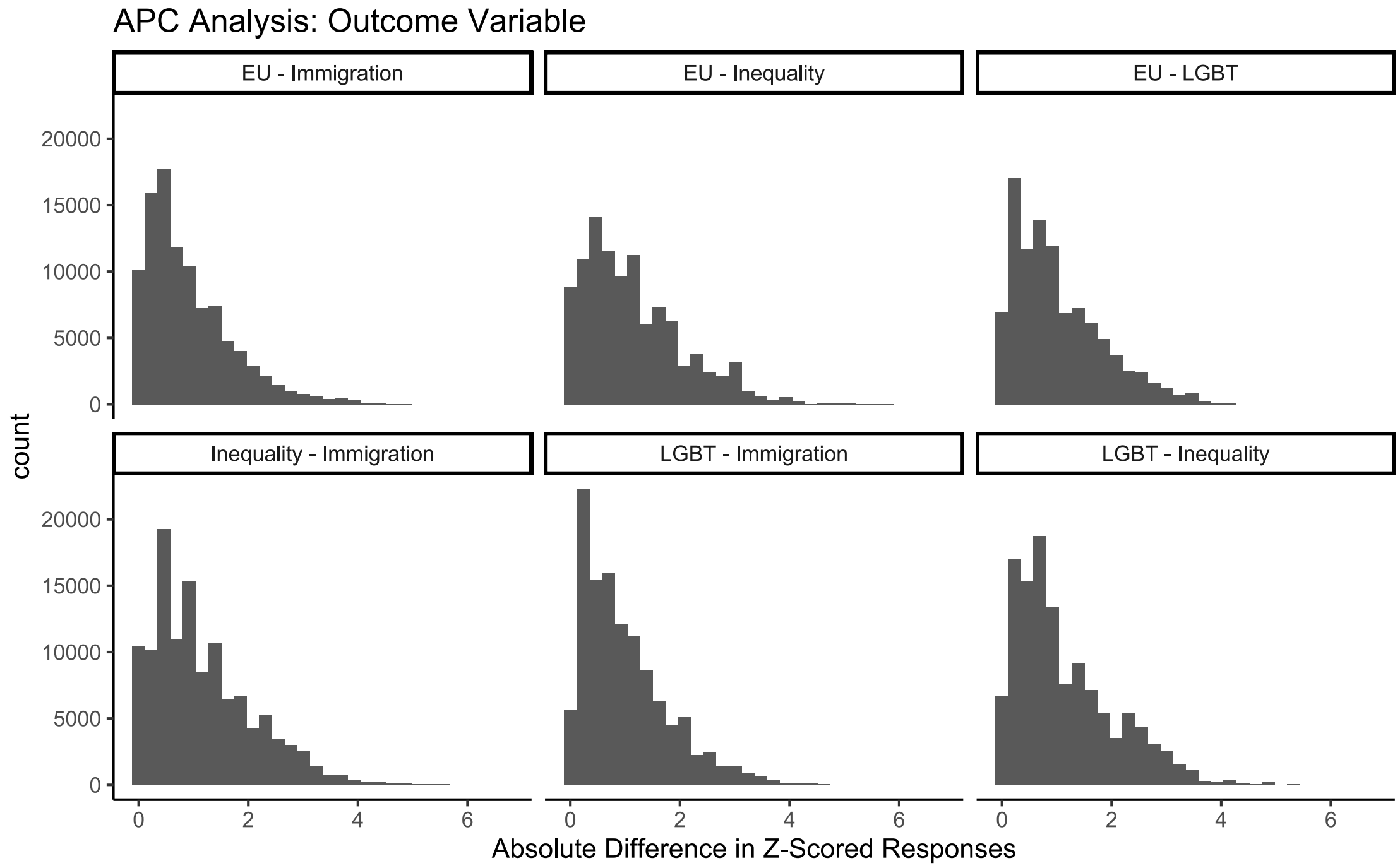

Figure C1: *Outcome Variable Description*

D. Models in Tabular Form

Figure 2A

Table D1: Fixed Effects

Term	Estimate	Std. Error	t value	p value
Intercept	1.093	0.014	77.510	< 0.001
Cohorts: Post-Transition	-0.023	0.003	-8.353	< 0.001
Cohorts: Post-EU	-0.040	0.005	-8.321	< 0.001
Gender (Woman)	-0.045	0.002	-18.194	< 0.001
Education	0.002	0.001	1.351	0.177
Income	-0.002	0.001	-3.664	< 0.001

Table D2: Approximate significance of smooth terms

Smooth Term	edf	Ref.df	F	p value
s(survey_year): Pre-Transition	8.478	8.928	18.880	< 0.001
s(survey_year): Post-Transition	8.379	8.897	29.620	< 0.001
s(survey_year): Post-EU	4.218	5.198	36.240	< 0.001
pair RE: BG	5.718	6.000	57.430	< 0.001
pair RE: CZ	5.900	6.000	217.970	< 0.001
pair RE: EE	5.904	6.000	213.900	< 0.001
pair RE: HR	5.763	6.000	49.160	< 0.001
pair RE: HU	5.852	6.000	131.510	< 0.001
pair RE: LT	5.895	6.000	183.880	< 0.001
pair RE: PL	5.853	6.000	151.260	< 0.001
pair RE: SI	5.751	6.000	81.990	< 0.001
pair RE: SK	5.870	6.000	113.030	< 0.001

Figure 2B

Table D3: Fixed Effects

Term	Estimate	Std. Error	t value	p value
Intercept	1.103	0.043	25.842	< 0.001
Gender (Woman)	-0.045	0.002	-18.173	< 0.001
Education	0.001	0.001	0.836	0.403
Income	-0.003	0.001	-5.112	< 0.001

Table D4: Approximate significance of smooth terms

Smooth Term	edf	Ref.df	F	p value
Smooth Term	edf	Ref.df	F	p value

Smooth Term	edf	Ref.df	F	p value
s(survey_year): Pre-Transition BG	5.852	5.990	19.966	< 0.001
s(survey_year): Post-Transition BG	5.770	5.977	19.566	< 0.001
s(survey_year): Post-EU BG	4.068	4.737	17.045	< 0.001
s(survey_year): Pre-Transition CZ	1.010	1.020	79.420	< 0.001
s(survey_year): Post-Transition CZ	5.003	6.006	13.589	< 0.001
s(survey_year): Post-EU CZ	2.824	3.483	5.046	0.001
s(survey_year): Pre-Transition EE	6.411	6.878	15.876	< 0.001
s(survey_year): Post-Transition EE	5.470	6.269	2.754	0.007
s(survey_year): Post-EU EE	4.938	5.797	8.685	< 0.001
s(survey_year): Pre-Transition HR	4.850	4.988	16.408	< 0.001
s(survey_year): Post-Transition HR	4.579	4.918	17.384	< 0.001
s(survey_year): Post-EU HR	4.014	4.635	2.958	0.010
s(survey_year): Pre-Transition HU	7.637	8.452	6.455	< 0.001
s(survey_year): Post-Transition HU	6.982	7.954	13.585	< 0.001
s(survey_year): Post-EU HU	3.497	4.304	3.978	0.002
s(survey_year): Pre-Transition LT	4.075	4.933	11.318	< 0.001
s(survey_year): Post-Transition LT	6.026	6.697	29.071	< 0.001
s(survey_year): Post-EU LT	3.726	4.513	15.304	< 0.001
s(survey_year): Pre-Transition PL	7.563	8.496	11.856	< 0.001
s(survey_year): Post-Transition PL	6.405	7.565	12.351	< 0.001
s(survey_year): Post-EU PL	7.220	8.236	13.900	< 0.001
s(survey_year): Pre-Transition SI	7.684	8.574	6.036	< 0.001
s(survey_year): Post-Transition SI	7.553	8.487	5.114	< 0.001
s(survey_year): Post-EU SI	2.445	3.038	2.763	0.040
s(survey_year): Pre-Transition SK	6.196	6.783	10.297	< 0.001
s(survey_year): Post-Transition SK	3.822	4.636	14.994	< 0.001
s(survey_year): Post-EU SK	1.767	2.203	2.898	0.059
pair RE	4.996	5.000	1154.354	< 0.001

Figure 3

Table D5: Fixed Effects

Term	Estimate	Std. Error	t value	p value
Intercept	1.136	0.010	112.193	< 0.001
Cohorts: Post-Transition	-0.027	0.004	-7.229	< 0.001
Cohorts: Post-EU	-0.077	0.006	-12.958	< 0.001
Gender (Woman)	-0.045	0.003	-18.099	< 0.001
Education	0.002	0.001	1.196	0.232
Income	-0.002	0.001	-3.691	< 0.001

Table D6: Approximate significance of smooth terms

Smooth Term	edf	Ref.df	F	p value
s(survey_year): euftf_freehms, Pre-Transition	8.903	8.994	24.278	< 0.001
s(survey_year): euftf_gincdif, Pre-Transition	8.823	8.981	16.636	< 0.001
s(survey_year): euftf_imwbcnt, Pre-Transition	8.990	9.000	137.571	< 0.001
s(survey_year): freehms_gincdif, Pre-Transition	6.422	7.594	10.420	< 0.001
s(survey_year): freehms_imwbcnt, Pre-Transition	7.231	8.275	15.541	< 0.001
s(survey_year): gincdif_imwbcnt, Pre-Transition	6.997	8.095	8.705	< 0.001
s(survey_year): euftf_freehms, Post-Transition	8.930	8.997	24.403	< 0.001
s(survey_year): euftf_gincdif, Post-Transition	8.897	8.993	27.162	< 0.001
s(survey_year): euftf_imwbcnt, Post-Transition	8.988	9.000	116.675	< 0.001
s(survey_year): freehms_gincdif, Post-Transition	4.153	5.121	15.377	< 0.001
s(survey_year): freehms_imwbcnt, Post-Transition	7.256	8.289	8.086	< 0.001
s(survey_year): gincdif_imwbcnt, Post-Transition	7.390	8.384	29.466	< 0.001
s(survey_year): euftf_freehms, Post-EU	3.136	3.886	9.766	< 0.001
s(survey_year): euftf_gincdif, Post-EU	1.925	2.393	48.220	< 0.001
s(survey_year): euftf_imwbcnt, Post-EU	4.184	5.123	24.635	< 0.001
s(survey_year): freehms_gincdif, Post-EU	5.128	6.216	42.306	< 0.001
s(survey_year): freehms_imwbcnt, Post-EU	3.988	4.916	7.044	< 0.001
s(survey_year): gincdif_imwbcnt, Post-EU	5.810	6.935	44.618	< 0.001
Country RE	7.832	8.000	38.294	< 0.001

E. Heterogeneity in Cohort Differences: Random Forest

To describe heterogeneity in generational differences across the nine studied countries, we turn to random forest, a machine-learning technique that enables us to disentangle potential reasons why generational differences appear among some individuals but not others. We take advantage of the European Social Survey data, which include numerous relevant covariates—many of which vary significantly across generations. As the share of tertiary-educated individuals has risen, younger cohorts tend to be more secular, more dissatisfied with politics, and, given their younger age, earn less; gender differences have also increased among these cohorts. The covariates considered include educational attainment, household income, belonging to a religious organization, level of religiosity, sex, and interest in politics.

Given the large number of observations, which makes the analysis computationally demanding, we focus on the youngest generation, socialized after EU accession, and how it differs from other generations. We train the model on the data using assignment to generations (1 for post-EU, 0 for others) as the main predictor and the aforementioned covariates. Random forest models are fit with 500 trees. Generational differences are predicted for each unit using out-of-bag predictions.

All conditional effects are summarized in Figure *8*. Given the large number of units (*386,796*), we binned effects into 1,000 equal-sized categories to display them effectively. In 75.4% of cases, there is no significant generational difference, while in a small subset (2.5%, upper tail), those belonging to the post-EU generation are even more disaligned in their beliefs. In a non-negligible subset (*22.1%*, lower tail), alignment in the post-EU generation is higher than in previous generations, consistent with our expectations.

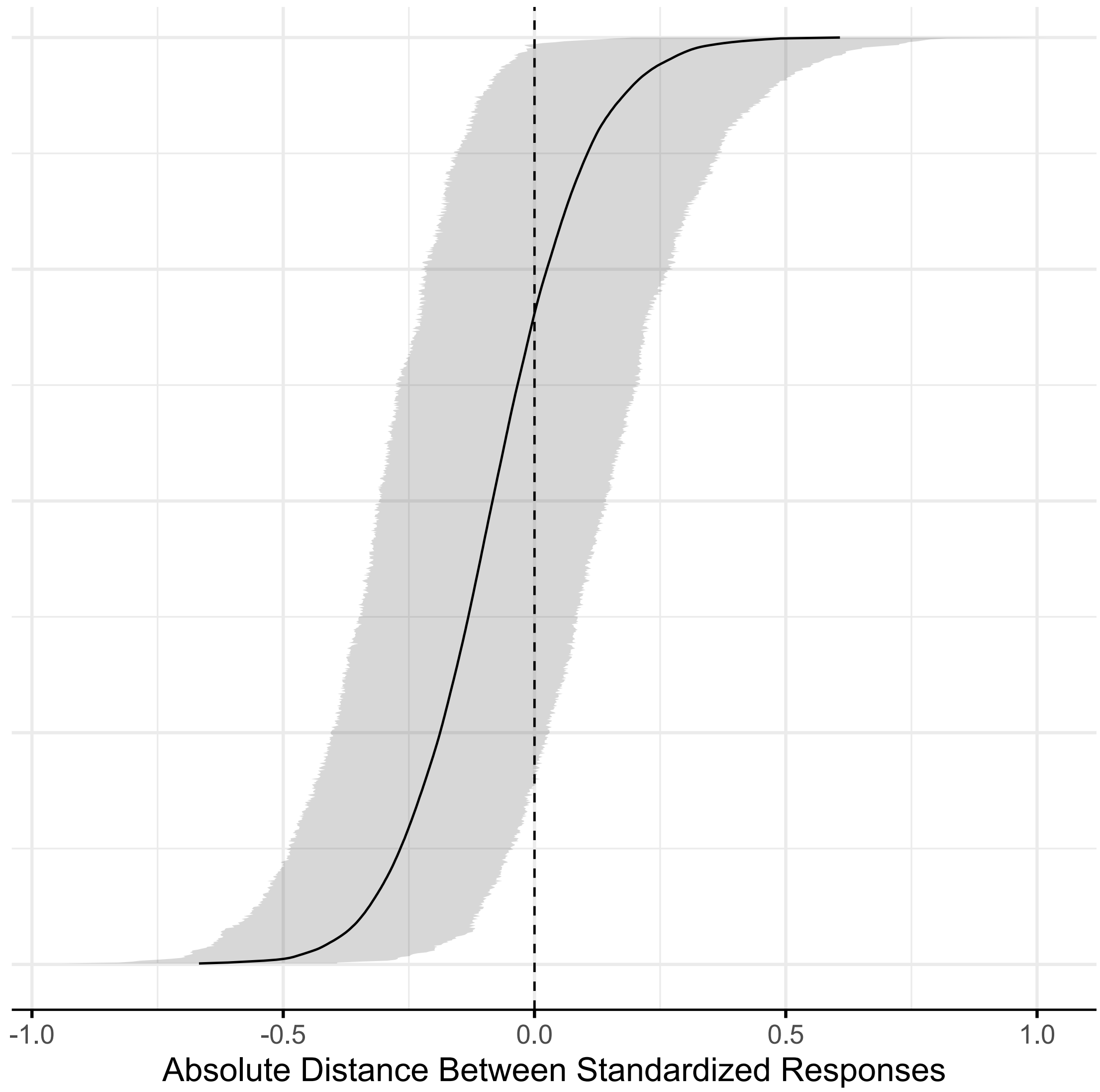

Figure E1: *Random Forest: Heterogeneity in Treatment Effects*

The model not only describes the extent of generational differences but also identifies which covariates are most important in driving this heterogeneity. This importance is presented in Figure 9. The measure should be interpreted only in relative terms across categories, as it mainly reflects how often a variable is used in tree splits; its absolute values carry little meaning.

First, we observe that educational attainment, more than any other variable, plays the largest role in explaining generational differences. This is unsurprising, as tertiary enrollment nearly doubled in many countries between 2004 and 2022 (e.g., Bulgaria: 44% to 84%; Croatia: 44% to 81%; data: *World Bank*), altering beliefs of younger generations. Second, household income and belonging to a religious organization are also important sources of heterogeneity, though to a lesser extent. Individual religiosity, sex, and interest in politics play only marginal roles.

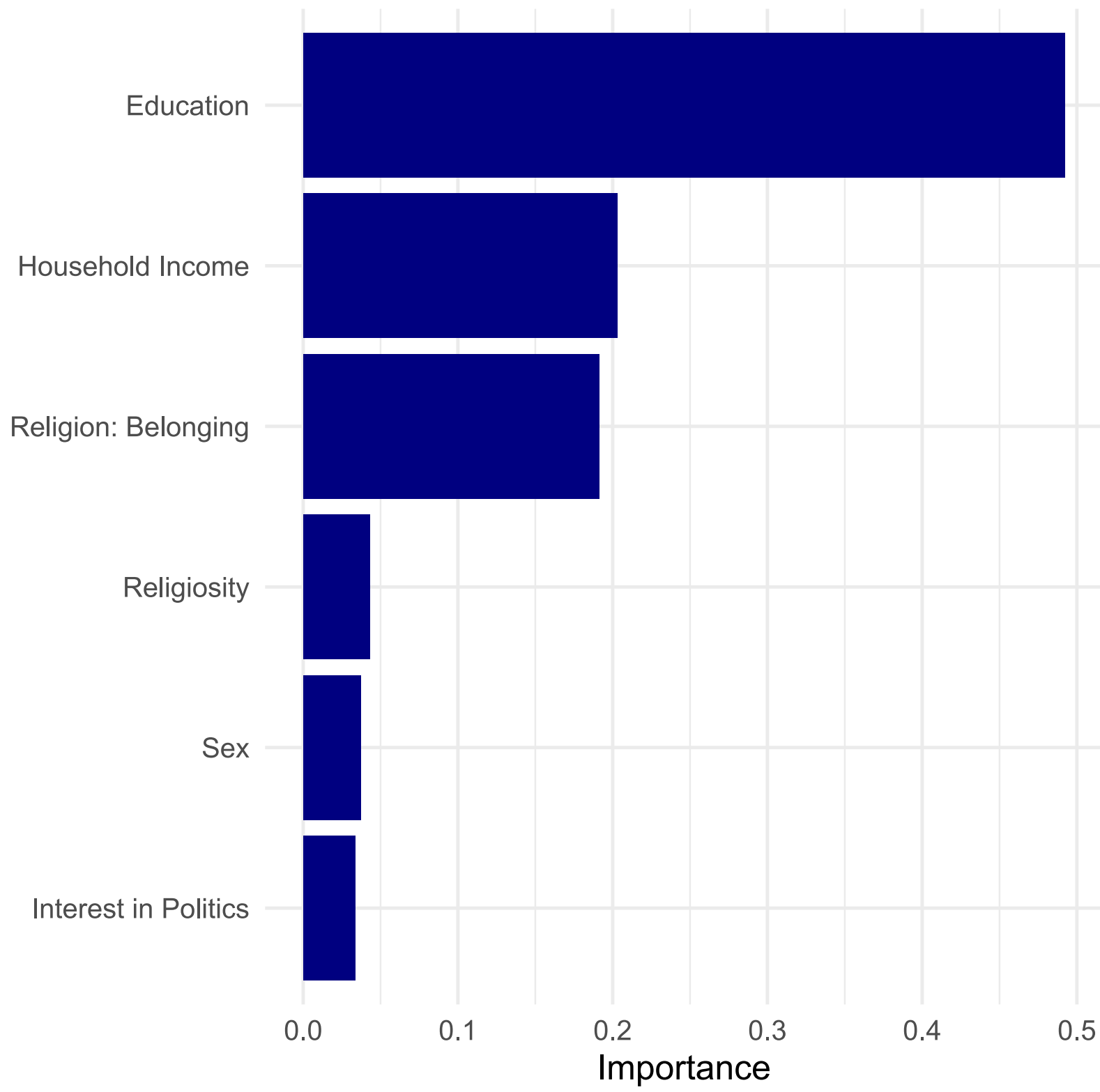

Figure E2: *Variable Importance: Post-EU Generation Compared to Others*

F. Centrality Measures for Belief Networks

To assess the centrality of individual nodes (i.e., political issues), we employ **node strength** as a centrality measure. Node strength is computed as the sum of the absolute values of edge weights—specifically, the partial correlations between each node and all others to which it is connected. To account for differences in network size, we use standardized z-scores of node strength, facilitating comparability across studies on similar topics. Formally, node strength centrality is defined as:

$$z_i = \frac{s_i - \mu_s}{\sigma_s}$$

where:

- z_i denotes the standardized strength of node i,
- $s_i = \sum_{j \in N(i)} \left| w_{ij} \right|$ represents the raw strength of node i,
- μ_s is the mean strength across all nodes,
- σ_s is the standard deviation of node strengths.

Why Strength as a Measure of Centrality?

Several altertive centrality metrics exist, including *closeness* (the average distance of a node from all other nodes), *betweenness* (the frequency with which a node lies on the shortest path between other nodes), and *expected influence* (similar to strength but incorporates the direction of associations). We opt for node strength due to its intuitive interpretability. Unlike expected influence, strength considers the magnitude of associations regardless of direction—aligning with our interest in capturing the relative importance of political issues, irrespective of whether associations between stances are ideologically consistent (positive) or inconsistent (negative).

An overview of the centrality of all issues in our data collection for each generation and country is provided in Figure *10*. Issues are organized in descending order of centrality. A first noteworthy observation is that aid to Ukraine is one of the most central issues across cohorts in all studied countries. Although subsequent positions vary across countries, economic and environmental issues tend to be the most isolated from other social issues overall (with Lithuania again standing out, as the welfare question ranks relatively high). Lastly, several intriguing generational differences emerge, particularly regarding the centrality of gender-related issues (with the exception of the item on male leaders in Czechia).

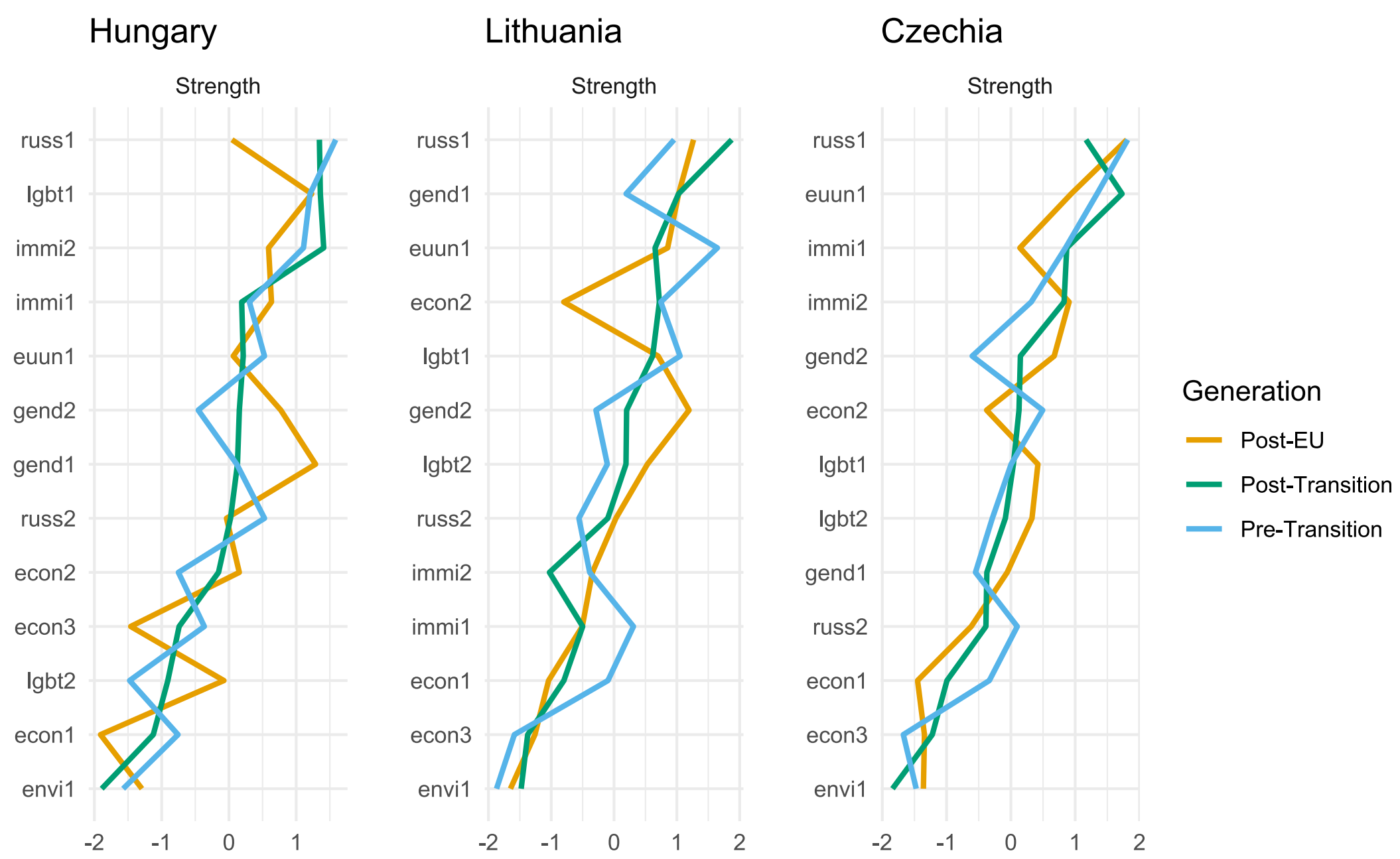

Figure F1: *Centrality of Issue Across Countries and Generations*

G. Belief Networks - Edge Weights

For further investigation, we present all the edge weights, represented visually in the network graphs and numerically in tables. Individual weights can be interpreted as partial correlations between opinion vectors on the issues in question (e.g., *European Union accession was a mistake* and *Immigration benefits the economy*). Consistent with the visualizations presented in the main paper, and to avoid unnecessary clutter, we report only those correlations that differ significantly from zero.

Czechia: Post-EU

	econ1	econ2	econ3	gend1	gend2	lgbt1	lgbt2	euun1	immi1	immi2	envi1	russ1	russ2
econ1													
econ2	0.08												
econ3	0.08	0.2											
gend1													
gend2				0.34									
lgbt1		0.03		0.17	0.12								
lgbt2				0.05	0.18	0.32							
euun1					0.07		0.12						
immi1		-0.08						0.16					
immi2	-0.11	-0.09		0.06		0.03		0.18	0.17				
envi1					-0.03				0.04	0.02			
russ1		-0.05			0.02	0.08	0.03	0.18	0.23	0.17	0.21		
russ2		-0.01	-0.02		0.05		0.03	0.17		0.04		0.16	

Czechia: Post-Transition

	econ1	econ2	econ3	gend1	gend2	lgbt1	lgbt2	euun1	immi1	immi2	envi1	russ1	russ2
econ1													
econ2	0.22												
econ3	0.15	0.22											
gend1													
gend2	0.02		-0.05	0.35									
lgbt1				0.16									
lgbt2			-0.04	0.07	0.18	0.3							
euun1		-0.18			0.1	0.03	0.08						
immi1		-0.01				0.08		0.14					
immi2	-0.06	-0.04	-0.03			0.05	0.02	0.16	0.33				
envi1	-0.01		0.01		-0.08	0.09			0.05	0.03			
russ1		-0.1						0.26	0.32	0.09	0.11		
russ2	-0.08			0.1	0.01	0.06	0.03	0.17		0.11		0.11	

Czechia: Pre-Transition

	econ1	econ2	econ3	gend1	gend2	lgbt1	lgbt2	euun1	immi1	immi2	envi1	russ1	russ2
econ1													
econ2	0.32												
econ3	0.09	0.14											
gend1													
gend2		-0.06		0.32									
lgbt1				0.11	0.05								
lgbt2					0.1	0.29							
euun1	-0.01	-0.2	-0.01			0.02							
immi1		-0.07			0.02	0.08	0.04	0.14					
immi2	-0.15			0.03				0.09	0.3				
envi1						0.04	0.11	0.07	0.01	0.02			
russ1	-0.03			0.02		0.06		0.35	0.28	0.21	0.05		
russ2	-0.02	0.04	-0.04	0.08		0.06	0.1	0.17			0.03	0.18	

Hungary: Post-EU

	econ1	econ2	econ3	gend1	gend2	lgbt1	lgbt2	euun1	immi1	immi2	envi1	russ1	russ2
econ1													
econ2													
econ3		0.21											
gend1	-0.08	0.1											
gend2		0.1		0.36									
lgbt1		0.09	0.03	0.15	0.1								
lgbt2				0.1	0.04	0.28							
euun1		0.05			0.26	0.08							
immi1		-0.04			-0.09	0.03							
immi2		-0.16		0.03		0.06	0.15	0.09	0.3				
envi1				-0.07		0.04			0.14				
russ1			0.01			0.16		0.08	0.31	0.09	0.08		
russ2	-0.07	0.02	0.04	0.2		0.06	0.11	0.19		0.02			

Hungary: Post-Transition

	econ1	econ2	econ3	gend1	gend2	lgbt1	lgbt2	euun1	immi1	immi2	envi1	russ1	russ2
econ1													
econ2	0.12												
econ3	0.05	0.33											
gend1	-0.07		0.1										
gend2		0.04	0.01	0.49									
lgbt1	-0.02	0.09		0.07	0.07								
lgbt2	-0.02		-0.03	0.02		0.27							
euun1		0.01	0.03		0.14	0.06	0.1						
immi1		-0.07			-0.04	0.11	0.01						
immi2	-0.11	-0.07		0.01	0.04	0.07	0.07	0.17	0.4				
envi1			0.05		-0.01				0.06				
russ1	-0.01		0.04			0.19		0.22	0.15	0.1	0.26		
russ2	-0.15	0.04		0.06		0.15	0.07	0.12		0.08		0.13	

Hungary: Pre-Transition

	econ1	econ2	econ3	gend1	gend2	lgbt1	lgbt2	euun1	immi1	immi2	envi1	russ1	russ2
econ1													
econ2	0.05												
econ3		0.29											
gend1													
gend2			0.03	0.46									
lgbt1		0.03	0.05	0.15	0.02								
lgbt2	-0.03					0.17							
euun1			0.01	0.02		0.13							
immi1	-0.03	-0.02				0.06		0.07					
immi2	-0.11		-0.03		0.02	0.07		0.18	0.42				
envi1			0.03					0.04	0.07				
russ1	-0.09		0.05			0.21		0.28	0.09	0.15	0.06		
russ2	-0.12	0.05	0.07	0.08		0.15	0.02	0.11		0.01		0.22	

Lithuania: Post-EU

	econ1	econ2	econ3	gend1	gend2	lgbt1	lgbt2	euun1	immi1	immi2	envi1	russ1	russ2
econ1													
econ2	0.15												
econ3	0.11	0.02											
gend1													
gend2		0.02		0.49									
lgbt1		-0.03											
lgbt2		-0.06		0.12	0.02	0.27							
euun1		0.01		0.03	0.25		0.01						
immi1						0.11	0.04						
immi2		-0.09		0.05	0.03	0.03	0.14		0.14				
envi1						0.1			0.07				
russ1	0.07		0.15			0.16		0.14	0.1		0.03		
russ2				0.09				0.3				0.17	

Lithuania: Post-Transition

	econ1	econ2	econ3	gend1	gend2	lgbt1	lgbt2	euun1	immi1	immi2	envi1	russ1	russ2
econ1													
econ2	0.32												
econ3	0.13	0.18											
gend1		-0.05	-0.03										
gend2	0.02	0.02		0.54									
lgbt1	-0.08	-0.1	-0.01	0.02									
lgbt2	-0.09	-0.08	-0.03	0.13		0.37							
euun1		-0.03			0.18								
immi1	-0.04	-0.08				0.08		0.03					
immi2		-0.01	-0.07	0.06			0.15	0.09	0.27				
envi1			0.04		-0.05	0.18			0.08				
russ1		-0.04	0.09			0.07		0.31	0.12		0.2		
russ2		-0.02		0.15	0.03			0.27	-0.03			0.29	

Lithuania: Pre-Transition

	econ1	econ2	econ3	gend1	gend2	lgbt1	lgbt2	euun1	immi1	immi2	envi1	russ1	russ2
econ1													
econ2	0.27												
econ3	0.12	0.15											
gend1													
gend2				0.48									
lgbt1	-0.09	-0.16		0.06									
lgbt2	-0.05	-0.06	-0.06		0.03	0.34							
euun1	-0.05	-0.11		0.13	0.03	0.01	0.04						
immi1	-0.08	-0.02				0.14	0.05	0.12					
immi2		-0.08		0.03		0.06	0.03		0.33				
envi1				0.02				0.08	0.02	0.03			
russ1	-0.01	-0.01				0.04		0.36	0.01	0.04	0.12		
russ2				0.01	0.08	0.02		0.12		0.01		0.32	

H. ESS: Inter-Item Alignment Descriptives

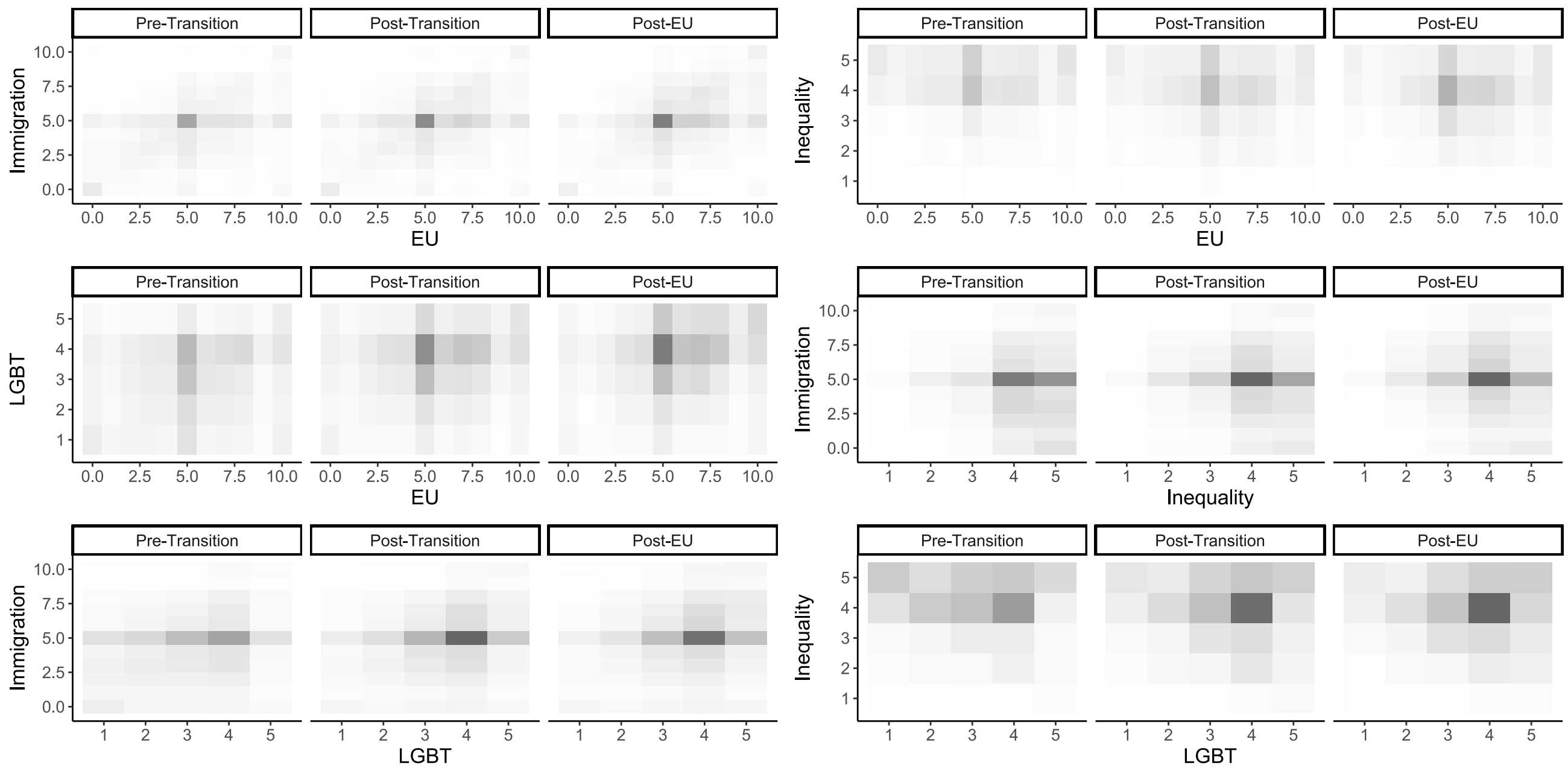

Figure H1: *Two-Dimensional Opinion Distributions For Each Issue Pair*
Note: Given the different sample sizes for each cohort, we depict sample shares within each tile of this plot. Higher values reflect left-wing / progressive attitudes.
